# (Pro)renin receptor mediates tubular epithelial cell pyroptosis in diabetic kidney disease via DPP4-JNK pathway

**DOI:** 10.1186/s12967-023-04846-5

**Published:** 2024-01-05

**Authors:** Shiying Xie, Shicong Song, Sirui Liu, Qiong Li, Wei Zou, Jianting Ke, Cheng Wang

**Affiliations:** 1 Division of Nephrology, Department of Medicine, The Fifth Affiliated Hospital Sun Yat-Sen University, Zhuhai, 519000 Guangdong China; 2 Guangdong Provincial Engineering Research Center of Molecular Imaging Center, The Fifth Affiliated Hospital Sun Yat-Sen University, Zhuhai, 519000 Guangdong China

**Keywords:** (Pro)renin receptor, Diabetic kidney disease, Renal tubular cell pyroptosis, Dipeptidyl peptidase 4

## Abstract

**Background:**

(Pro)renin receptor (PRR) is highly expressed in renal tubules, which is involved in physiological and pathological processes. However, the role of PRR, expressed in renal tubular epithelial cells, in diabetic kidney disease (DKD) remain largely unknown.

**Methods:**

In this study, kidney biopsies, urine samples, and public RNA-seq data from DKD patients were used to assess PRR expression and cell pyroptosis in tubular epithelial cells. The regulation of tubular epithelial cell pyroptosis by PRR was investigated by in situ renal injection of adeno-associated virus9 (AAV9)-shRNA into *db/db* mice, and knockdown or overexpression of PRR in HK-2 cells. To reveal the underlined mechanism, the interaction of PRR with potential binding proteins was explored by using BioGrid database. Furthermore, the direct binding of PRR to dipeptidyl peptidase 4 (DPP4), a pleiotropic serine peptidase which increases blood glucose by degrading incretins under diabetic conditions, was confirmed by co-immunoprecipitation assay and immunostaining.

**Results:**

Higher expression of PRR was found in renal tubules and positively correlated with kidney injuries of DKD patients, in parallel with tubular epithelial cells pyroptosis. Knockdown of PRR in kidneys significantly blunted *db/db* mice to kidney injury by alleviating renal tubular epithelial cells pyroptosis and the resultant interstitial inflammation. Moreover, silencing of PRR blocked high glucose-induced HK-2 pyroptosis, whereas overexpression of PRR enhanced pyroptotic cell death of HK-2 cells. Mechanistically, PRR selectively bound to cysteine-enrich region of C-terminal of DPP4 and augmented the protein abundance of DPP4, leading to the downstream activation of JNK signaling and suppression of SIRT3 signaling and FGFR1 signaling, and then subsequently mediated pyroptotic cell death.

**Conclusions:**

This study identified the significant role of PRR in the pathogenesis of DKD; specifically, PRR promoted tubular epithelial cell pyroptosis via DPP4 mediated signaling, highlighting that PRR could be a promising therapeutic target in DKD.

**Supplementary Information:**

The online version contains supplementary material available at 10.1186/s12967-023-04846-5.

## Background

Diabetic kidney disease (DKD) is the primary cause of end-stage renal disease (ERSD) and is associated with high morbidity and mortality rates [1]. Currently, a number of potential drugs showed beneficial effects in mouse model of DKD such as dipeptidyl peptidase 4 (DPP4) inhibitor, sodium-glucose cotransporter-2 inhibitors, glycolysis inhibitors, mineralocorticoid antagonists, inhibitors of renin-angiotensin system and peptide AcSDKP [2–10]. Furthermore, novel pathways have been proved play significant roles in aggravating the progression of DKD such as TGFβ signaling, WNT signaling, Notch signaling and Hedgehog signaling, or providing protective effect in halting DKD process such as endothelial glucocorticoid receptor, FGFR1 and SIRT3-mediated mechanisms in mouse model of DKD [4, 9, 11–13]. The pathogenic mechanisms driving DKD is under further exploration based on these vital and novel findings [1, 14]. Due to the histological changes observed at early stages of DKD, pathological injury of the glomerulus was the focus of previous studies [14, 15]. Notably, recent evidence showed that tubular epithelial cell (TEC) injury was occurred at an early stage of DKD, preceding even the onset of microalbuminuria [16]. Moreover, accumulating evidence have proved the role of injured TECs in DKD, including promoting renal inflammatory response, interstitial fibrosis and impairing tubular function [17, 18]. Therefore, blocking the injuries of TECs is a promising therapeutic strategy for DKD.

Pyroptosis, a gasdermin-mediated programmed cell death, is dependent on the NLRP3 (NOD-, LRR-, and pyrin-domain containing protein 3) inflammasome-regulated autoproteolysis of Caspase 1 and characterized by plasma membrane rupture and pro-inflammatory cytokine release [19]. Caspase-mediated cleavage of gasdermin liberates the pore-forming domain to disrupt the cell membrane and triggers pyroptosis [2]. Gasdermin D (GSDMD) and Gasdermin E (GSDME) are the well-characterized members of gasdermin family. GSDMD is the substrate of pro-inflammatory caspases (Caspase 1, 4, 5, and 11), while GSDME could be cleaved by Caspase 3 and killer cell-released granzyme B under certain stress. [20, 21]. Pyroptosis is reported to be implicated in the pathogenesis of acute kidney injury and chronic kidney disease by recent studies [22–24]: deletion of GSDME alleviated ureteral obstruction-induced renal tubular injury, inflammation and fibrosis [20]; blocking the pyroptotic death of endothelial cell or tubular cell suppressed inflammatory response and halted the process of DKD [22, 25]. Although emerging studies have demonstrated the pivotal role of pyroptosis in DKD, the precise mechanisms remain obscure.

The renin-angiotensin system (RAS) plays a vital role in the pathogenesis of DKD and RAS inhibition with antagonists is a commonly used therapeutic strategy to slow DKD progression [26, 27]. (Pro)renin receptor (PRR) is a novel component of RAS which could activate local RAS via the nonproteolytic activation of prorenin and regulate intracellular signal transduction in a RAS-independent manner [28]. In the kidney, PRR is enriched in renal tubules and highly involved in physiological and pathological processes, including mediating fluid balance, electrolyte homeostasis, renal inflammation, and fibrotic responses [29–31]. Multiple studies, using genetic and pharmacological approaches, have demonstrated the regulation of inflammatory response by PRR: deletion of PRR reduced ocular inflammatory molecules expression and macrophage infiltration in diabetic mice, while overexpression of PRR markedly increased macrophage infiltration and proinflammatory cytokine level in abdominal aortic aneurysm [32–34]. Despite the fact that augmentation of inflammatory response by PRR was observed, the underlying mechanisms remain elusive. Furthermore, the role of PRR and underlying molecular mechanisms in TECs of DKD patients need further exploration.

Dipeptidyl peptidase 4 (DPP4) is a pleiotropic serine peptidase and increases blood glucose by degrading incretins under diabetic conditions [35]. The primary structure of DPP4 consists of an intracellular tail (amino acids 1 – 6), transmembrane region (amino acids 7 – 28), flexible stalk (amino acids 29 – 47), glycosylated region (amino acids 48 – 323), cysteine-enrich region (amino acids 325 – 551) and catalytic region (amino acids 552 – 766) [36]. Crystal structure of human DPP4 from Protein Data Bank revealed that there are mainly two domains composed of the protein: highly conserved α/β-hydrolase domain and extra-enzymatic activities associated β-propeller [37]. The β-propeller is open and consist of glycosylation-rich and cysteine-rich subdomains [37]. Studies have elucidated adenosine deaminase (ADA) and caveolin-1, bind to the glycosylation-rich subdomain whereas other molecules including collagen, fibronectin, plasminogen and streptokinase bind to the cysteine-rich region [36]. As novel type of anti-diabetic agents, DPP4 inhibitors have showed beneficial effects in DKD in clinical and experimental studies [35, 38]. Meanwhile, the upregulation of renal tubular DPP4 was also observed in human and experimental rodents under diabetic conditions [35, 39]. Beyond glucose metabolism, the potential effect of DPP4 inhibitors as anti-inflammatory agents has been suggested by a variety of studies due to the significant reduction of plasma inflammation indicators and infiltration of immune cells in different tissues, indicating the regulation of DPP4 on inflammatory response [39, 40]. Furthermore, studies have proved that resident fibroblasts generated by epithelial-mesenchymal transition (EMT) and endothelial-mesenchymal transition (EndMT) accelerate the pathological process of DKD by promoting kidney fibrogenesis [3, 8]. As a key regulator of EMT and EndMT, DPP4 may contribute to DKD by promoting kidney fibrosis via suppressing SIRT3, FGFR1 and glucocorticoid receptor which are widely investigated anti-EMT and Anti-EndMT molecules [4, 8, 12, 41–45].

In this study, we confirmed the upregulation of PRR and occurrence of tubular cell pyroptosis by using renal biopsy specimens and urine samples of DKD patients. We further demonstrated that PRR promoted tubular epithelial cell pyroptosis to aggravate DKD via DPP4/JNK signaling. In summary, the present study provided new insights into the role of PRR in DKD, which may be helpful in halting the progression of DKD.

## Methods

### Human kidney biopsies and urine samples

All human samples (kidney biopsies or urine) were collected from patients at The Fifth Affiliated Hospital Sun Yat-Sen University following provision of written consent. The inclusion and exclusion criteria of the DKD patients were listed as follows: Male or female patients aged ≥ 18 years old with type 2 diabetes defined by the American Diabetes Association in the 2010 Standards of Medical Care in Diabetes [46], and biopsy-proven diabetes kidney disease with the new pathologic classification provided by the Renal Pathology Society [47] was included in this research. Key exclusion criteria included patients with significant non-diabetic kidney disease, renal arteries stenosis, uncontrolled hypertension (SBP ≥ 170 mmHg or DBP ≥ 110 mmHg), chronic heart failure with persistent symptoms (New York Heart Association class II – IV), using DPP4 inhibitor. The 5 renal samples were obtained from the patients who underwent tumor nephrectomies without diabetes or kidney disease were used as normal control. The 16 kidney biopsies samples were obtained from type 2 diabetes patients with biopsy-proven diabetic kidney disease (DKD), accompanied by clinical syndrome characterized by persistent albuminuria (> 300 mg/24 h) [1]. The demographic and clinical data of DKD patients are presented in Additional file 2: Table S1. For urine samples, per 50 ml of the clean morning urine samples were collected from 28 type 2 diabetes patients with biopsy-proven DKD and 23 healthy subjects during routine physical examination. The demographic and clinical data of DKD patients and health subjects are presented in Additional file 2: Table S2. All the human sample investigations were approved by the Research Ethics Committee of The Fifth Affiliated Hospital, Sun Yat-Sen University (No.: 2022#K180-1).

### Bioinformatics analysis

We used the published dataset (the GEO series number was GSE30529) [48], which detected the gene level of dissected tubules of kidney biopsies from DKD patients, from the NCBI Gene Expression Omnibus. We downloaded the gene expression matrix from supplementary file on the website and included the genes expressed in all samples for analysis. GSEA was used to analysis gene differential expression between different groups.

The interaction of PRR and DPP4 was predicated by BioGRID databases (https://thebiogrid.org/).

### Animal models

Male diabetic *db/db* mice (C57BLKS/J-LepR*db/db*) and their age-matched nondiabetic wild-type littermates (C56BLKS/J background) were purchased from GemPharmatech (Nanjing, China). We used *db/db* mice with C57BLKS/J background as type II diabetes nephropathy model because C57BLKS/J is not only permissive with hyperglycemia but also associated with more prominent kidney injury than other substrain. All the animal experiments were approved by the Institutional Animal Care and Use Committee of the Sun Yat-Sen University School of Medicine (No. 00292).

### In situ injection of adeno-associated virus to kidneys

AAV9 carrying PRR interference sequences (AAV9-shPRR, 1 × 10^12^ copies/ml) or the negative control were established by Genechem company (Shanghai, China). The surgery procedure was performed following the instruction of previous study [49]. To deliver the vector, 33G, 50 μl Hamilton syringes (7803-05, 7655-01, Hamilton Company) were used for multi-point injection around the renal cortex region. Briefly, the wildtype (WT) mice and *db/db* mice were anesthetized and maintained by isoflurane. The mice laid prone on a heating pad to keep the body temperature during the procedure. An incision (approximately 1 cm) was made in the right posterior to expose the right kidney. Injections were performed by carefully piercing into the kidney cortex, then slowly injecting with 10 μl of vector at one site. After injection and needle withdrawal, a dry cotton swab was used for 1 min for hemostasis and fluid leakage oppression. According to the manufacturer’s instruction, either AAV9-shPRR or AAV9-shNC was administered at 50 μl volumes of vector per mouse. The five selected sites were shown in Additional file 1: Fig. S1. Briefly, the five injection sites were located on the middle position of both sides of kidney, the upper pole of kidney, the middle position of the outer edge of kidney, and the lower pole of kidney. Mice were infected at 8 weeks, and blood and tissue samples were collected 3 months after AAV treatment.

**Fig. 1:**
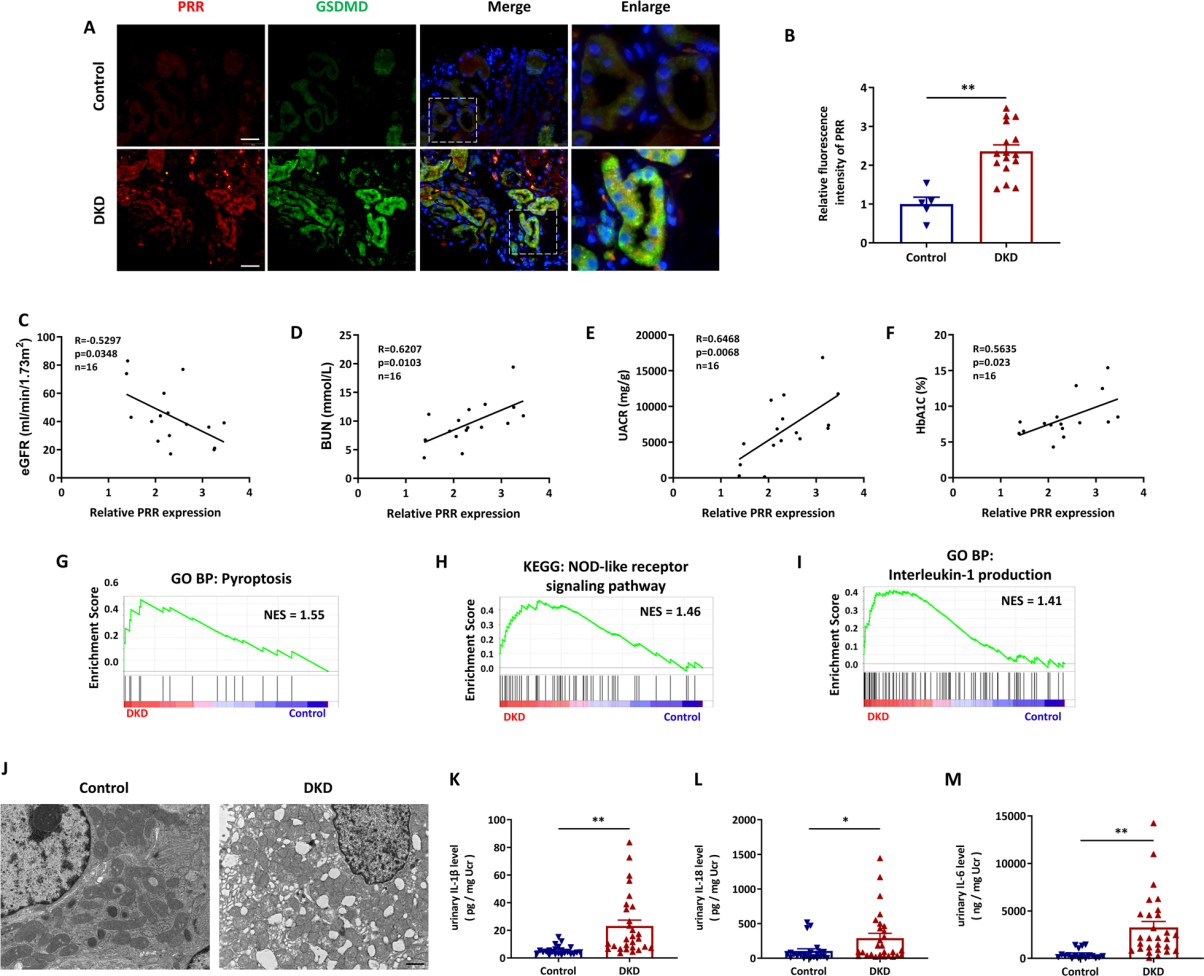
PRR expression and cell pyroptosis were increased in renal tubules of DKD patients. **A** and **B** Representative fluorescent images of coimmunostaining PRR (red) with GSDMD (green) in kidney biopsies from DKD patients (n = 16), and the control subjects were from paracancerous tissue (n = 5) (**A**), and quantitative data showed the upregulation of PRR in renal tubule. Bar = 20 μm. (**B**). **C**–**F** Analysis of the association between relative tubular PRR expression and eGFR(**C**), BUN (**D**), urinary albumin-to-creatinine ratio (UACR) (**E**) and Hemoglobin A1c (HbA1c) (**F**) of patients with DKD. (**G**–**I**) GSEA analysis of pyroptosis (**G**), NOD-like receptor signaling pathway (**H**) and Interleukin-1 production (**I**) in renal tubules of DKD patients (n = 10) and healthy controls (n = 12) by using public GSE dataset (GSE30529). **J** Representative microscopy images of tubular cell morphology in renal sections from DKD patients and health controls. Bar = 1 μm. (**K** and **L**) Elisa detection of urinary IL-1β (**K**) and IL-18 (**L**) from DKD patients (n = 28) and health controls (n = 23). **M** Elisa detection of urinary IL-6 from DKD patients (n = 28) and health controls (n = 18). Data are presented as mean ± SEM of biologically independent samples. ∗P < 0.05, ∗∗P < 0.01. *P* values were determined by Student’s t-test for comparison between two groups

### Examination of renal function

Urinary albumin was measured using an ELISA kit (Exocell, Cat#1011) and the values were normalized with urinary creatinine levels. The urinary creatinine was detected by a urinary creatinine assay kit (Exocell, Cat#1012) followed the manufacturer’s instruction. The urine albumin excretion rate was calculated as the urine albumin/creatinine ratio (UACR). Serum urea nitrogen or serum creatinine was measured using a Urea Nitrogen Colorimetric Detection Kit (Applygen, Cat#E2020) or creatinine detection Kit (Bioassay Systems, Cat#DICT-500) according to the manufacturer’s instructions.

### PAS staining

The staining was conducted following the manufacturer’s protocol (Leagene Biotech, Cat#DG0005). The tissue sections were deparaffinized and rehydrated by running the slices from xylenes to ethanol and water. The slices were oxidized in 0.5% periodic acid solution for 8 min and rinsed in tap water for 5 min. Then the slices were immersed in the Schiff reagent for 15 min until the sections became light pink color. Hematoxylin was applied to the slices for counterstain. Dehydrate and cleaning were conducted in sequence before coverslip. Assessment of the mesangial and glomerular cross-sectional areas was performed by pixel counts in a blinded fashion. Tubular injury was scored in the cortical region on a scale from 0 to 4, based on tubular dilatation, loss of brush border and tubular structure and tubular atrophy: 0 (normal); 1 (less than 25%); 2 (25–50%); 3 (50–75%); and 4 (more than 75%).

### Sirius red staining

The staining was conducted following the manufacturer’s protocol (Leagene Biotech, Cat#DC0041). Briefly, deparaffinized sections were incubated with picrosirius red solution for 1 h at room temperature. The slides were washed with distilled water. Then the slides were subjected to Mayer hematoxylin stain solution for 8 min. After wash with distilled water, dehydrate and cleaning were conducted in sequence before coverslip to the slides. Sirius red staining was imaged and analyzed using the Image J software, and the fibrotic areas were quantified.

### Masson staining

The staining was conducted following the manufacturer’s protocol (Leagene Biotech, Cat#DC0033). The tissue sections were deparaffinized and rehydrated by running the slices from xylenes to ethanol and water. The slices were stained in Weigert’s iron hematoxylin working solution for 5 min, and then rinsed the slices with tap water. Then stained the slices in Biebrich scarlet-acid fuchsin solution for 10 min, followed by differentiation with phosphomolybdic–phosphotungstic acid solution for another 10 min. Aniline blue solution was subjected to the slices directly after differentiation followed by acid alcohol for 10 s. Dehydrate and cleaning were conducted in sequence before coverslip. The histological fibrosis was measured according to the following scoring system: 0 = no positive area; 1 = 1 – 10% positive area; 2 = 11 – 25% positive area; 3 = 26 – 50% positive area; 4 ≥ 51% area.

### Immunohistochemistry

Immunohistochemistry was carried out as previous described [50, 51]. Briefly, fresh tissues were fixed by formalin and then fully dehydrated and paraffin embedded. The paraffin blots were cut into 5 – 10 μm sections. Antigen retrieval was performed using citrate buffer (10 mM, Solarbio Life Sciences, Cat# C1032) by autoclaving at 120 ℃ for 10 min. Endogenous peroxidase was quenched by 1% H_2_O_2_ for 10 min. After blotting with 10% donkey serum, primary antibodies were applied to the slices at 4 ℃ overnight, follow with biotinylated secondary antibodies incubation for 1 h at room temperature, and then StreptAvidin—BiotinComplex for another 1 h (Boster, Cat#BA1088). Primary antibodies dilutions list as follow, F4/80 1:10,000 (Cell Signaling Technology, Cat#70076;); Kim-1:10,000 (Invitrogen, cat# MA5-28211), WT-1 (Cell Signaling Technology, Cat#83535), CD31 (Abcam, Cat#ab76533). DAB was applied to visualize the antibodies labelling and hematoxylin was applied to the slices for counterstain. Immunohistochemistry of KIM-1 was quantified using Image J software. Briefly, the color threshold of the images was adjusted in RGB mode. Then we drag the bars (0–255 grey scale) under each color to modify the selection. In our case, brown staining was selected. Once the binary image was done, clicked the Analyze Particles to get the value for analyzation.

### Immunofluorescence

The fixation, dehydration, paraffin embedding and antigen retrieval were performed as previously described [50]. After blotting with donkey serum, the slices were applied to primary antibodies at 4 ℃ overnight. Then the specific secondary antibodies were applied 1 h at room temperature. Primary antibodies dilutions list as follow, PRR 1:200 (Sigma-Aldrich, Cat#SAB2702080); GSDMD 1:200 (Abcam, Cat#ab209845); DPP4 1:200 (Santa Cruz, Cat#sc-52642); Ly6G 1:500 (Abcam, Cat#238132). The nuclei were counter-stained with DAPI for imaging. We used Image J software to quantify the immunofluorescence staining. Briefly, we separated different channels of images by clicking Image-color-Split channels, then we captured the considered signal by color thresholding. During this procedure, we scrolled through the options in the menu to make sure the green/red pixels were above the threshold and were counted. Then we clicked the area integrated intensity and mean grey value boxes to get the fluorescence intensity value. The same parameters were used to analyze the images.

### Transmission electron microscope and scanning electron microscopy

Samples were imaged respectively by a transmission electron microscope (TEM) (JEM-1400PLUS, Japan) and scanning electron microscopy (SEM) (Thermo Fisher Scientific, Phenom Pure, USA). For TEM analyses, the fresh renal tissues were rapidly fixed in 2.5% glutaraldehyde. After washing in 0.1 M phosphate buffer, the renal tissues were fixed for 1 h in 1% osmic acid. The osmicated samples were then dehydrated through a series of graded series of ethyl alcohol concentrations. After that, the samples and then embedded and sliced, followed by staining uranyl acetate (3%) and lead citrate. Subsequently, the sections were recorded by TEM. Thickness of the glomerular basement membrane (GBM) was analyzed using ImageJ software. For SEM analyses, the cells were fixed with 2.5% glutaraldehyde, rinsed in 0.1 M PBS for 45 min and postfixed in the dark for 2 h using 1%OsO_4_ at room temperature. After being dehydrated and dried, the specimens were mounted on stubes and sputter-coated with gold–palladium. The images were monitored by SEM.

### Cell culture and transfection

HK-2 cells, originally obtained from American Type Culture Collection (ATCC, Cat#CRL-2190), were maintained in DMEM/F12 supplemented with 10% FBS, 100 U/ml penicillin, and 100 mg/ml streptomycin at 37 ℃ in a humidified atmosphere of 5% CO2 [52]. HK-2 cells were stimulated with normal glucose (NG) at 5.6 mmol/L glucose plus 24.4 mM mannitol, and high glucose (HG) at 30 mmol/L glucose for 72 h. PRR and DPP4 siRNA and control siRNA were obtained from Genepharma, overexpression vector of PRR was generously provided by Professor Zhou Lili. The sequences were listed as follow: PRR-siRNA: 5ʹ-AGAAUAUUAAGUGGAAGUGGGUGAA-3ʹ; DPP4-siRNA: 5ʹ-GCAGUACCCAAAGACUGUATT- 3ʹ. N-terminal Flag-DPP4 (amino acids 1–323), C-terminal Flag-DPP4 (amino acids 325–766) and cysteine-enrich region of C-terminal Flag -DPP4 (amino acids 325–551) were cloned into the pcDNA3.0 vector (Tianyi Biological Technology Co., Ltd, Guangzhou, China). The HK-2 cells were transfected with siRNA or vector using Lipofectamine 3000 (Thermo Fisher Scientific, Cat#L3000015) according to the manufacturer’s protocol, and the cells were cultured for an additional 48 h before harvested. Losartan was used at the concentration of 10 μM (MCE, Cat#HY-17512).

### Immunoblotting and immunoprecipitation

Immunoblotting was performed as pervious described [53]. Firstly, we prepared lysis buffer by adding protease and phosphatase inhibitors (Biosharp Technology Co., Ltd, China). Then the tissues were homogenized in lysis buffer on ice. For the HK-2 cells, we added the lysis buffer to the plates and collected the lysate for centrifuge. The protein amounts were measured by ABC Kit (Beyotime Biotech. Inc. China). Equal amounts of protein were electrophoresed by sodium dodecyl sulfate–polyacrylamide (SDS-PAGE) gel and transferred to PVDF membranes (Millipore-Sigma, USA). The membranes were blotting with 5% nonfat milk for 1 h at room temperature followed by primary antibodies incubation at 4℃ overnight with their respective dilutions as follow, DPP4 1:1000 (Abcam, Cat#ab129060; Santa Cruz, Cat#sc-52642); cleaved-Caspase1 (CST, Cat#4199S, Santa Cruz, Cat# sc-398715), GSDMD-N 1:1000 (Abcam, Cat#ab215203; Abcam Cat#ab219800,), IL-18 1:1000 (Abcam, Cat#ab243091; Abcam, Cat#ab191860), IL-1β 1:1000 (Abcam, Cat# ab9722), NLRP3 1:1000 (Abcam, Cat# ab263899), p-JNK ((Abcam Cat#ab124956), JNK (Abcam Cat#ab179461), Flag (Sigma-Aldrich Cat#SAB4301135), PRR 1:1000 (Sigma-Aldrich, Cat#HPA003156), SIRT3 (Abcam, Cat#ab217319, Cat#Ab246522), FGFR1 (Proteintech, Cat#60325-1), E-cadherin (CST, Cat#3195S), CD31 (Abcam, Cat#ab76533), Vimentin (CST, Cat#5741 T), α-SMA (CST, Ca#48938S), Fibronectin (Abcam, Cat#ab2413), β-actin 1:10,000 (CST, Cat# 4970S). The HRP-conjugated goat anti-rabbit (Abcam, Cat#ab205718) or goat anti-mouse (Abcam, Cat#ab205719) secondary antibodies were applied to the membranes after several washed with TBST. Signal was captured using the SuperSignal West Femto Maximum Sensitivity Substrate kit (ThermoFisher Scientific, Cat#34095).

For coimmunoprecipitation and immunoprecipitation, HK-2 cells were lysed with chilled NP-40 lysis buffer containing protease and phosphatase inhibitors. Protein concentration of the supernatant was measured as previously described. The Protein A/G beads (ThermoFisher Scientific, Cat#88802) was washed for 3 times and then incubated with primary antibody (PRR, 1:100) or IgG at room temperature for 1 h. After the incubation, the beads were washed for 3 times and incubated with the same amount of cell lysates at 4 ℃ overnight. On the next day, the beads were washed with IP buffer and the elution buffer was subjected to the beads to elute the lysates for immunoblotting**.**

### IL-1β, IL-18 and IL-6 ELISA

Concentrations of urinary IL-1β or IL-18 of DKD patients were measured using a human IL-1β ELISA kit (ThermoFisher Scientific, Cat#88-7261-22), human IL-18 ELISA kit (Abcam, Cat#ab215539) or human IL-6 ELISA kit (ThermoFisher Scientific, Cat#88-7066-22) according to the manufacturer’s protocols, the concentrations of urinary IL-1β, IL-18 or IL-6 were calculated by urinary creatinine. The concentrations of urinary IL-1β or IL-18 of mice were determined by using an IL-1β mouse ELISA kit (ThermoFisher Scientific, Cat#88-7013-22) or IL-18 mouse ELISA kit (RayBio, Cat#elm-IL18-1) according to the manufacturer’s protocols, the concentrations of urinary IL-1β or IL-18 were normalized by urinary creatinine concentration. The concentration of IL-1β, IL-18 or IL-6 in the culture medium of HK-2 cells was detected by using human IL-1β ELISA kits (Cloud-Clone Corp, Cat#SEA563Hu), human IL-18 ELISA kits (Cloud-Clone Corp, Cat#SEA064Hu) or human IL-6 ELISA kits (ThermoFisher Scientific, Cat#88-7066-22) followed the manufacturer's protocols, the concentrations of medium IL-1β or IL-18 were normalized by total proteins of cell lysates.

### Caspase 1 activity measurement

Caspase 1 activity was measured by Caspase 1 activity assay kit according to the manufacturer's instructions (Beyotime Biotechnology, Cat#C1102). Briefly, the cell lysates were mixed with reaction buffer containing Ac-YVAD-ρNA and incubated at 37 ℃ for 2 h. The absorbance was detected at 405 nm with a microplate reader and the caspase 1 activity was normalized with total proteins of cell lysates.

### Flow cytometry analysis

Pyroptosis was measured by FAM-FLICA Caspase-1 Detection Kit (Immunochemistry Technologies, Cat#98). Briefly, the treated cells were harvested and stained with the FLICA and PI, then washed the cells with washing buffer and collected the cells for analysis using LSR Fortessa flow cytometers (BD Biosciences, USA).

### Statistical analyses

All quantitative data were presented as means ± standard errors (SEs). Student’s t-test was applied to two-group comparisons. One-way ANOVA was used for comparisons including more than two groups and Tukey’s post hoc test was used to estimate the significance between two groups once significant differences were found. Differences were considered statistically significant when P < 0.05. Correlation analysis was performed using Spearman (nonparametric) analysis, P < 0.05 was considered statistically significant. All statistical analysis were performed in GraphPad Prism software V8.

## Results

### PRR expression and cell pyroptosis were increased in renal tubules of DKD patients

To evaluate the role of (pro)renin receptor (PRR) in diabetic kidney disease (DKD), we first examined its expression in DKD patients. Compared with healthy controls, tubular PRR expression was highly induced in DKD patients, accompanied by the co-localization of increased gasdermin D (GSDMD) in tubules (Fig. 1A, B). Additionally, PRR expression was correlated with clinical indexes of patients, including: estimated glomerular filtration rate (eGFR) (Fig. 1C), blood urea nitrogen (BUN) (Fig. 1D), urine albuminuria creatinine ratio (UACR) (Fig. 1E) and Hemoglobin A1c (HbA1c) (Fig. 1F) suggesting the potential involvement of PRR in DKD. As GSDMD was significantly increased in tubules, we speculated that tubule epithelial cell pyroptosis occurred during DKD. This hypothesis was supported by the analysis of published GSE dataset (GSE30529), which detected the gene level of dissected tubules of kidney biopsies from DKD patients. The subsequent GSEA analysis revealed that pyroptosis (Fig. 1G), NOD-like receptor signaling (Fig. 1H), and interleukin (IL)-1 production (Fig. 1I) were activated in renal tubules of DKD patients. We also observed the morphological changes of tubular cells using transmission electronic microscope (TEM). Tubular cells of healthy control displayed a normal, healthy morphology, and the cytoplasm contained abundant mitochondria and endoplasmic reticulum. In comparison, the morphology of tubular cells from DKD patients showed severe ultrastructural disruptions, including organelle swelling and damage, chromatin condensation and cytoplasmic vacuolization (Fig. 1J). In the kidney of DKD patients, the pathological changes of glomerulus were also observed. Compared to controls, thicker glomerular basement membrane (GBM), less podocytes and decreased endothelial cell density were observed in DKD patients (Additional file 1: Fig. S2A–F). We also detected higher levels of urinary IL-1β (Fig. 1K), IL-18 (Fig. 1L) and IL-6 (Fig. 1M) in patients with DKD compared to the controls. These data indicated that pyroptosis was increased in renal tubules of patients with DKD and that PRR might be related to pyroptosis.

### Knockdown of PRR effectively blocked high glucose (HG)-augmented HK-2 cell pyroptosis

**Fig. 2:**
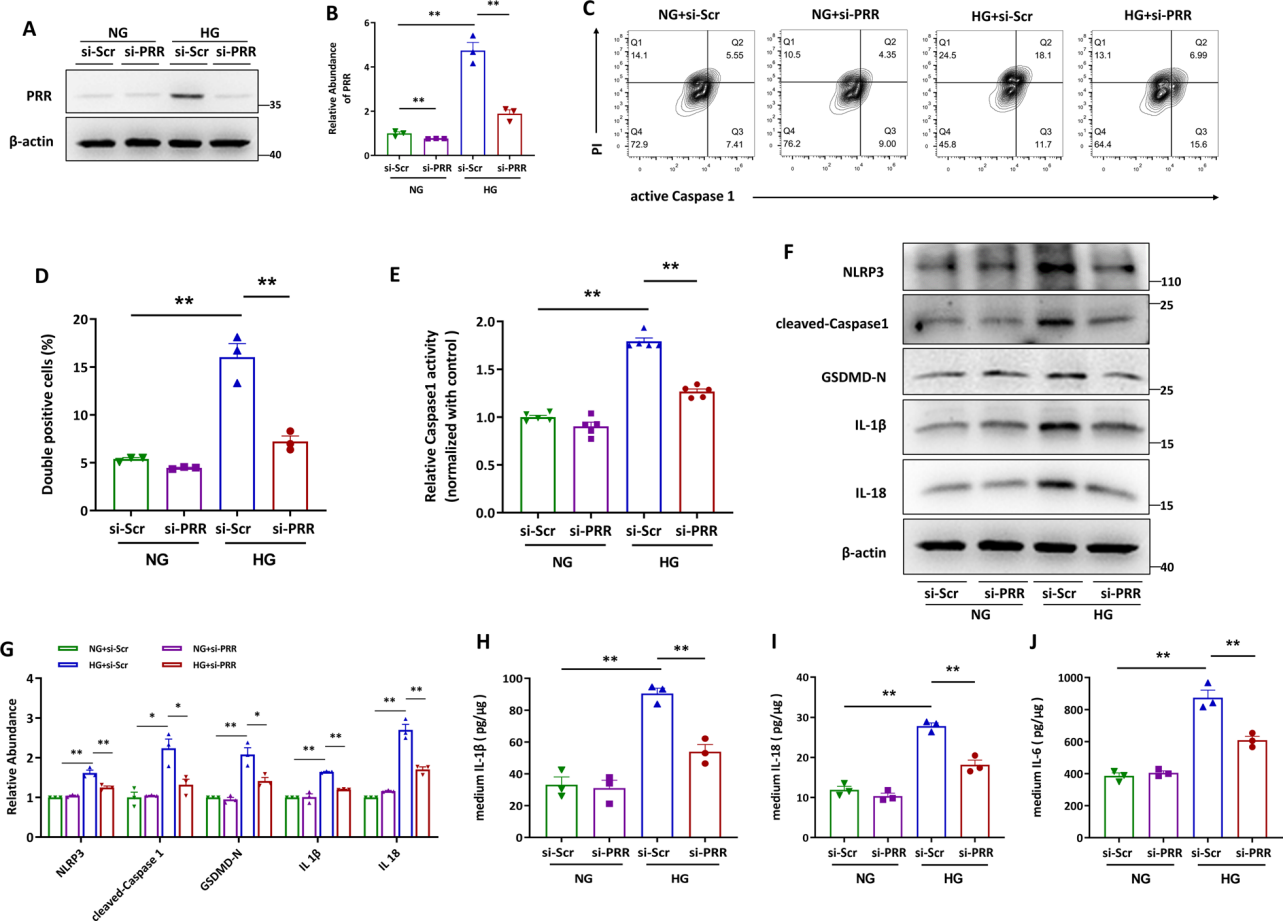
Knockdown of PRR effectively blocked high glucose (HG)-augmented HK-2 cell pyroptosis. **A** and **B** Western blot analyses (**A**) and quantitative data (**B**) showed that knocking down by siRNA diminished HG-induced PRR expression. (n = 3). **C** and **D** Flow cytometry analysis showed that HG induced active Caspase1^+^PI^+^ HK-2 cells were decreased by PRR siRNA (**C**) and quantitative data (**D**). (n = 3). **E** PRR was silenced in HG stimulated HK-2 cells, and the Caspase1 activity in cell lysis was determined by kits (n = 5). **F** and **G** Western blot analyses (**F**) and quantitative data (**G**) showed that PRR siRNA blocked HG-induced NLRP3, cleaved-Caspase1, GSDMD-N, IL-1β and IL-18 expression in HK-2 cells (n = 3). **H** and **I** PRR was ablated in HG treated HK-2 cells, and the IL-1β (H), IL-18 (I) or IL-6 (**J**) concentration in the culture medium was determined by ELISA, and then normalized by protein concentration in cell lysates (n = 3). Data are presented as mean ± SEM of biologically independent samples. ∗ P < 0.05, ∗∗ P < 0.01. One-way ANOVA was used to analyze the data among multiple groups, followed by Tukey’s post hoc test

Because increased PRR and pyroptotic cell death were detected in renal tubules (Fig. 1A, B), we thus investigated whether PRR exerted any effect on renal tubular cell pyroptosis via an in vitro study. Knockdown of PRR by siRNA effectively blocked HG-induced PRR expression (Fig. 2A, B), accompanied by decreased percentage of active Caspase 1 positive and propidium iodide (PI) positive HK-2 cells stimulated by HG (Fig. 2C, D), indicating that PRR promoted HG-induced HK-2 cell pyroptosis. As Caspase 1 plays a pivotal role in pyroptotic cell death by cleaving full length GSDMD to release its N-terminal domain (GSDMD-N), we subsequently performed experiments for detection of intracellular Caspase 1 activity. Accordingly, knockdown of PRR attenuated HG-induced Caspase 1 activation in HK-2 cells (Fig. 2E). As activation of the NOD-like receptor protein 3 (NLRP3) inflammasome, formation of cell membrane pore by GSDMD-N, and release of IL-1β and IL-18 are characteristics of pyroptosis, we examined the abundance of these proteins by immunoblotting. As expected, deleting PRR blocked HG-stimulated protein level of NLPR3, cleaved-Caspase1, GSDMD-N, IL-1β, and IL-18 (Fig. 2F–G). Similar trends were observed for the release of IL-1β, IL-18 and IL-6 by HG-treated HK-2 cells (Fig. 2H–J). Furthermore, PRR knockdown also alleviated the typical morphological changes of pyroptosis in HG-stimulated HK-2 cells (Additional file 1: Fig. S3). The above data suggested the promotion by PRR of HG-induced HK-2 cell pyroptosis.

### Overexpression of PRR promotes HK-2 cell pyroptosis independent of Angiotensin II

**Fig. 3:**
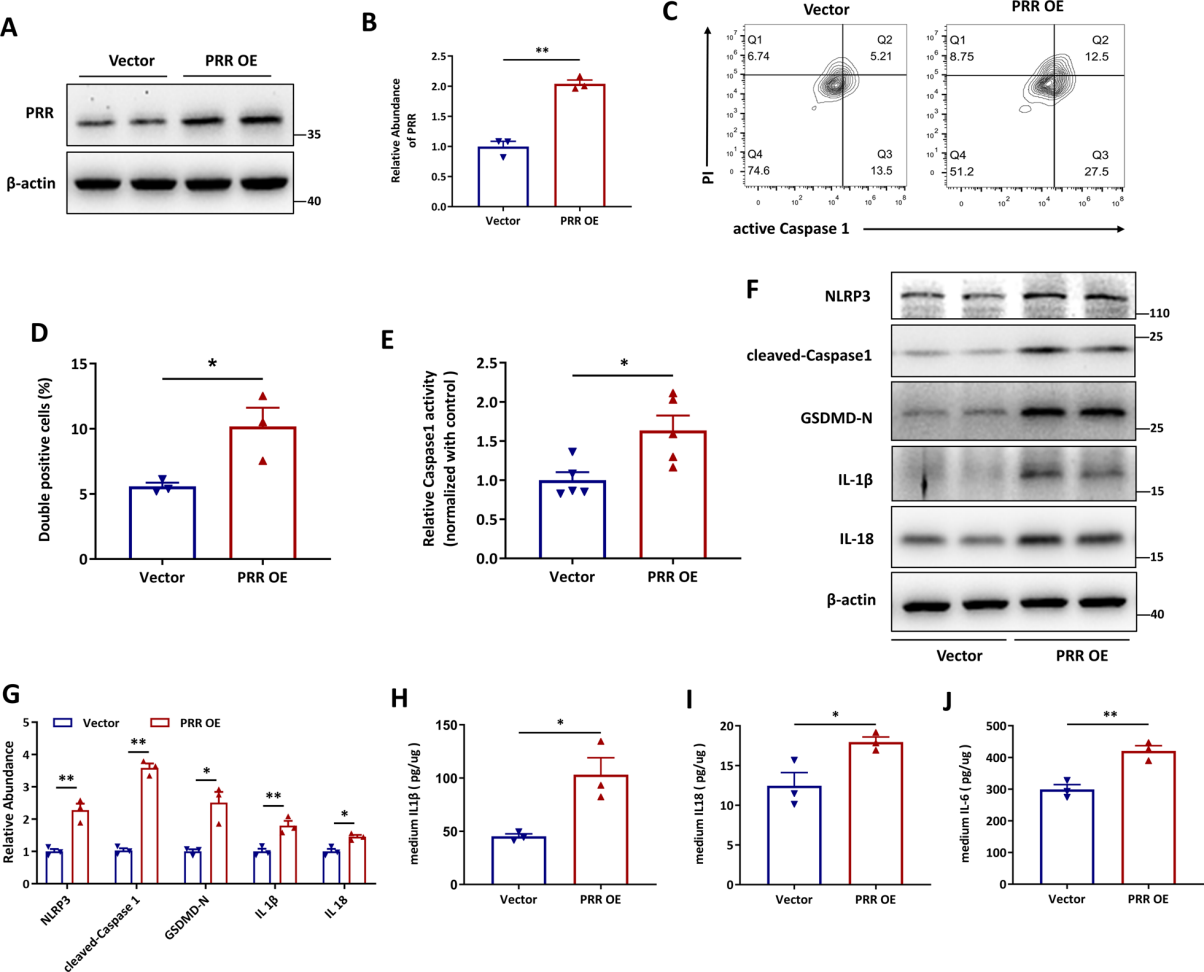
Overexpression of PRR promotes HK-2 cell pyroptosis. (**A** and **B**) Western blot analyses. **A** and quantitative data (**B**) showed the increased protein abundance of PRR in PRR overexpression plasmid transfected HK-2 cells. (n = 3). (**C** and **D**) Flow cytometry analysis showed that active Caspase1^+^PI^+^ HK-2 cells were increased by PRR overexpression (**C**) and quantitative data (**D**) (n = 3). **E** PRR was overexpressed in HK-2 cells, and the Caspase1 activity in cell lysis was determined by kits (n = 5). (**F** and **G**) Western blot analyses (**F**) and quantitative data (**G**) showed that PRR overexpression increased NLRP3, cleaved-Caspase1, GSDMD-N, IL-1β and IL-18 expression in HK-2 cells. (n = 3). **H** and **I** PRR was overexpressed in HK-2 cells, and the IL-1β (H), IL-18 (**I**) or IL-6 (**J**) concentration in the culture medium was determined by ELISA, and then normalized by protein concentration in cell lysis. (n = 3). Data are presented as mean ± SEM of biologically independent samples. ∗ P < 0.05, ∗∗ P < 0.01. *P* values were determined by Student’s t-test

To confirm the effect of PRR on pyroptotic cell death, PRR was overexpressed in HK-2 cells. As shown, PRR abundance (Fig. 3A, B), as well as the percentage of active Caspase 1 and propidium iodide (PI) labeled pyroptotic cells were highly induced by transfection of the expression vectors encoding PRR (Fig. 3C, D). Propidium iodide (PI) is a small fluorescent molecule that binds to DNA but cannot passively traverse into cells that possess an intact plasma membrane. As Caspase 1 activation resulted in the N-terminal of gasdermin oligomerization and pore formation in the plasma membrane, which subsequently leaded to the rupture of the plasma membrane and then pyroptotic cells are permeable to PI [54]. Hence, PI and Caspase 1 were used to label the pyroptotic cells. The upregulation of intracellular Caspase 1 activity was also observed in PRR-overexpressed HK-2 cells (Fig. 3E). Furthermore, overexpression of PRR augmented the abundance of NLRP3, cleaved Caspase1, GSDMD-N, IL-1β, and IL-18 in HK-2 cells (Fig. 3F–G) and increased the release of IL-1β, IL-18 and IL-6 (Fig. 3H–J). As PRR is an important component of local renin-angiotensin system (RAS), we detected the involvement of Angiotensin II in PRR regulated pyroptotic cell death in HK-2 cells. The results revealed that blocking Angiotensin II receptor with losartan showed no significant effect on PRR overexpression induced NLRP3, cleaved-Caspase1, GSDMD-N, IL-1β and IL-18 expression in HK-2 cells (Additional file 1: Fig. S4), indicating that PRR could regulate HK-2 cells pyroptosis independent of Angiotensin II.

### PRR interacted with DPP4 to activate JNK signaling and inhibit SIRT3 signaling and FGFR1 signaling in vitro

**Fig. 4:**
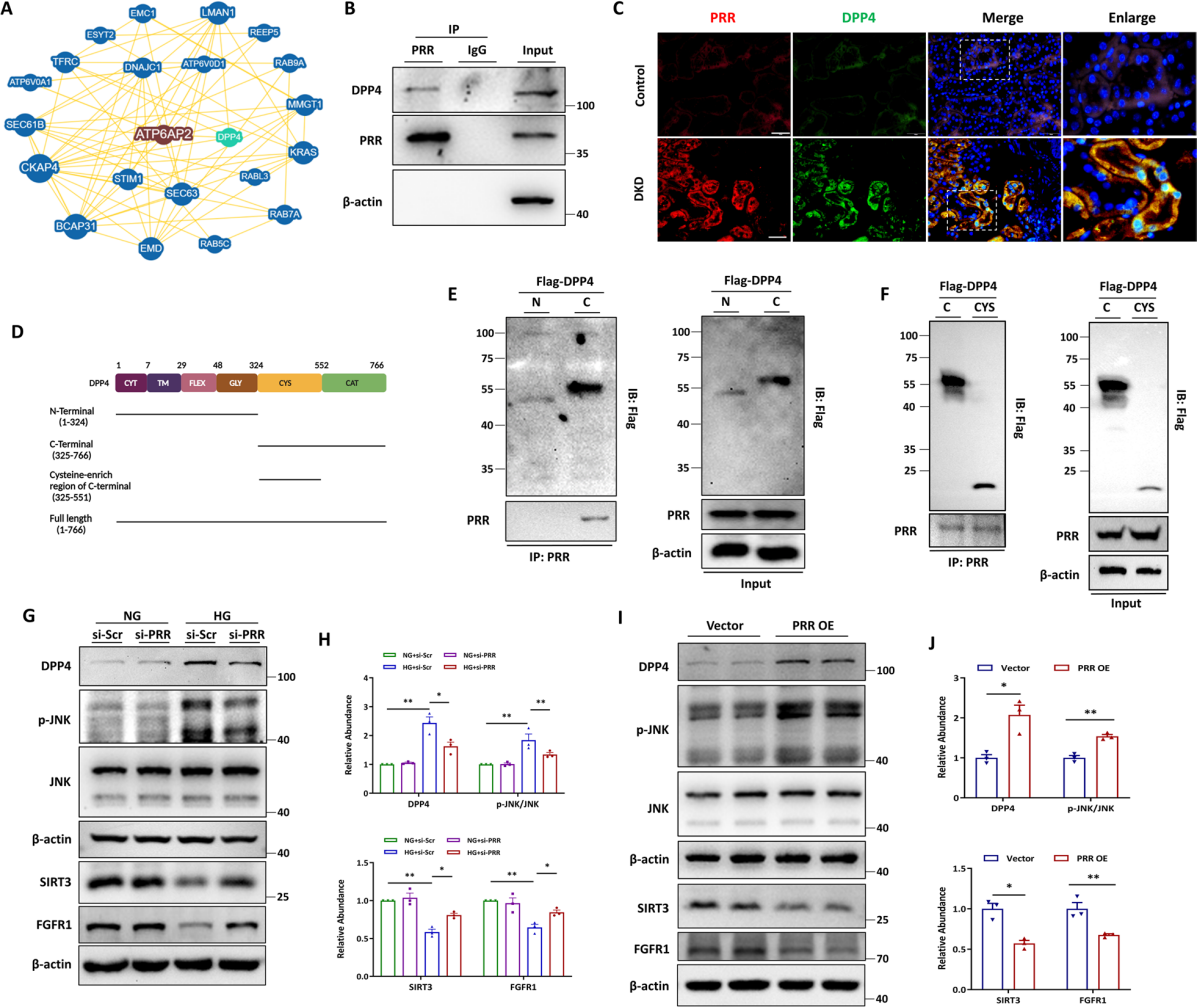
PRR interacted with DPP4 to activate JNK signaling and inhibit SIRT3 signaling and FGFR1 signaling in vitro*.* **A** BioGRID database was used to analyze the potential interaction between PRR and DPP4. **B** Co-immunoprecipitation (Co-IP) to detect the interaction between PRR and DPP4. The protein lysates isolated from HK-2 cells were immunoprecipitated with anti-PRR antibody and were immunoblotted with indicated antibodies. **C** Representative fluorescent images of coimmunostaining PRR (red) with DPP4 (green) in renal sections from DKD patients and the healthy control was from paracancerous tissue. Bar = 20 μm. **D** Schematic representation of the DPP4 domain. **E** Co-IP was used to detect the interaction between PRR and Flag-DPP4. HK-2 cells were transfected with Flag-tagged truncation of DPP4 C-terminal 325–766 amino acids (Flag-DPP4 C), or 1–324 amino acids (Flag-DPP4 N). Protein lysates were immunoprecipitated with anti-Flag antibody and immunoblotted with the indicated antibodies. **F** Co-IP was used to detect the interaction between PRR and Flag-DPP4. HK-2 cells were transfected with Flag-tagged truncation of DPP4 C-terminal 325–766 amino acids (Flag-DPP4 C), or 325–551 amino acids (Flag-DPP4 CYS). Protein lysates were immunoprecipitated with anti-Flag antibody and immunoblotted with the indicated antibodies. **G** and **H** Western blot analyses (**G**) and quantitative data (**H**) showed that PRR siRNA blocked HG-induced DPP4 abundance and phosphorylation of JNK, and restored HG-suppressed SIRT3 and FGFR1 expression in HK-2 cells (n = 3). **I** and **J** Representative Western blot (**I**) and quantitative data (**J**) showed increased DPP4, upregulated phosphorylation of JNK, suppressed SIRT3 and reduced FGFR1 in PRR overexpressed HK-2 cells (n = 3). Data are presented as mean ± SEM of biologically independent samples. ∗ P < 0.05, ∗∗ P < 0.01. One-way ANOVA was used to analyze the data among multiple groups, followed by Tukey’s post hoc test

BioGrid database [55] was used to analyze the protein which interacts with PRR. We found that dipeptidyl peptidase 4 (DPP4) was a potential binding partner of PRR (Fig. 4A). Co-immunoprecipitation (Co-IP) was performed to verify this interaction, and the analysis showed that endogenous PRR could bind DPP4 in HK-2 cells (Fig. 4B). Moreover, the upregulation and co-localization of PRR and DPP4 in renal tubules of DKD patients were detected by immunofluorescence staining (Fig. 4C), confirming the interaction of PRR and DPP4. Next, to analyze which domain of DPP4 interacted with PRR, we transfected the constructed DPP4-N-terminal (amino acids 1–324), DPP4-C-terminal (amino acids 325–766) and DPP4-C-terminal (cysteine-enrich domain, amino acids 325–551) plasmids into HK-2 cells (Fig. 4D). IP analyses showed the C-terminus of DPP4 binding with PRR, suggesting that PRR interacted with DPP4 through binding to the C-terminal of DPP4 (Fig. 4E). In order to figure out the specific domain interact with PRR, we constructed another plasmid expressing the recombinant cysteine-enrich region of C-terminal (amino acids 325–551). PRR antigen was detected in the flag-tagged-precipitation of cell lysis transfected with cysteine-enrich plasmid. The results showed that PRR bound to cysteine-enrich region of DPP4-C-terminal (Fig. 4F). We further investigated the effect of PRR on DPP4 expression in HK-2 cells. Intriguingly, PRR knockdown attenuated the HG-augmented protein level of DPP4 in HK-2 cells (Fig. 4G, H), while overexpression of PRR increased DPP4 protein level (Fig. 4I, J). Furthermore, a similar trend in DPP4 was observed for c-Jun N-terminal kinase (JNK), SIRT3 and FGFR1, reflected as reverse of HG-induced JNK phosphorylation and HG-suppressed SIRT3 and FGFR1 expression by PRR knockdown (Fig. 4G, H), and induced JNK activation and reduced SIRT3 and FGFR1 expression by PRR overexpression in HK-2 cells (Fig. 4I, J).

### DPP4 facilitated HG-induced HK-2 cell pyroptosis by activating JNK and suppressing SIRT3 and FGFR1

**Fig. 5:**
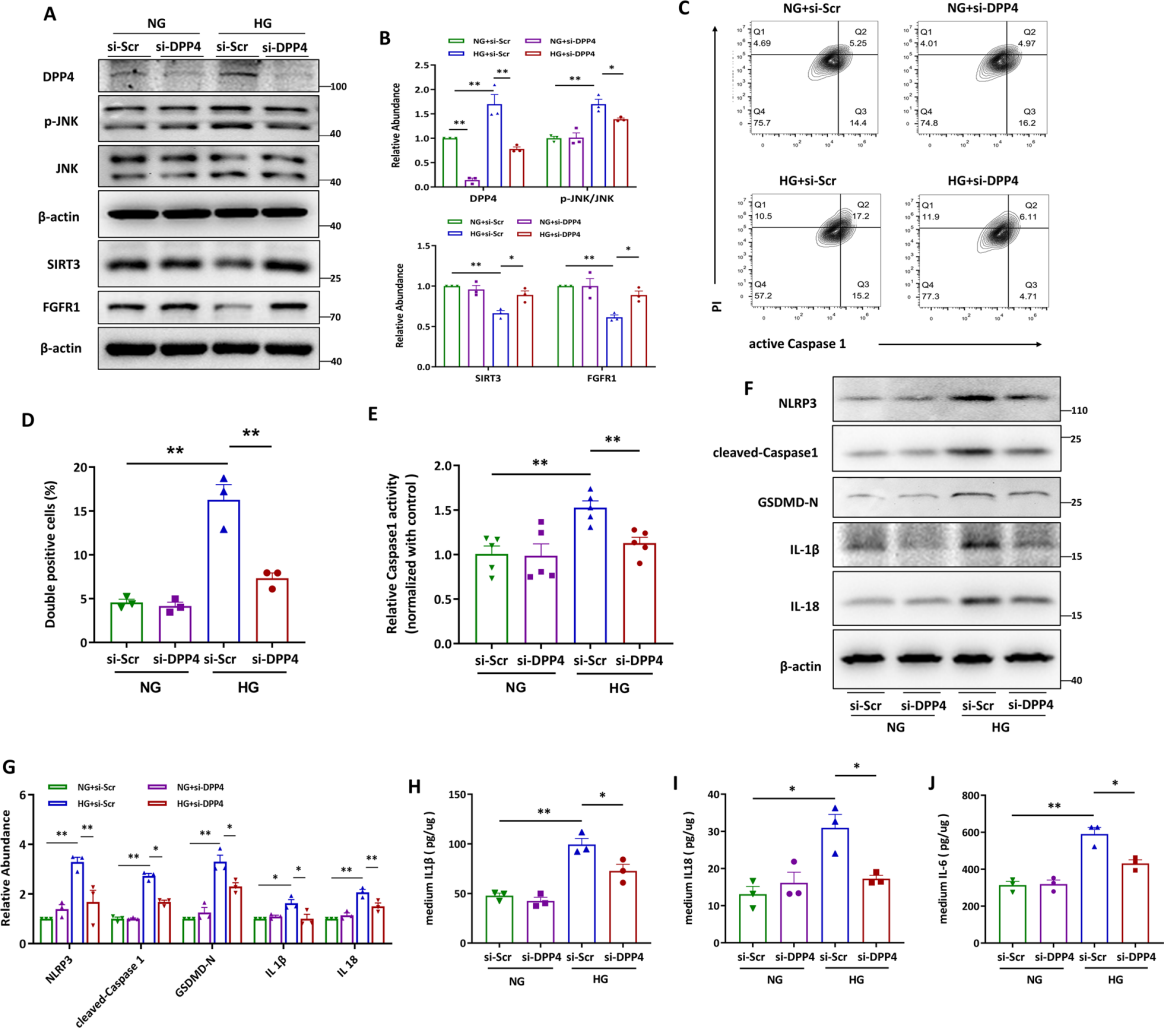
DPP4 facilitated HG-induced HK-2 cell pyroptosis by activating JNK and suppressing SIRT3 and FGFR1. **A** and **B** Western blot analyses. **A** and quantitative data (**B**) showed that knocking down by siRNA diminished HG-induced DPP4 expression and phosphorylated JNK, and restored HG-reduced SIRT3 and FGFR1 abundance. (n = 3). **C** and **D** Flow cytometry analysis showed that knocking down DPP4 reduced HG increased active Caspase1^+^PI^+^ HK-2 cells (**C**) and quantitative data (**D**) (n = 3). **E** DPP4 was ablated in HG stimulated HK-2 cells, and the Caspase1 activity in cell lysis was determined by kits. **F** and **G** Western blot analyses (**F**) and quantitative data (**G**) showed that DPP4 siRNA attenuated HG-induced NLRP3, cleaved-Caspase1, GSDMD-N, IL-1β and IL-18 expression in HK-2 cells (n = 3). (** H**and** I**) DPP4 was knocked down in HG treated HK-2 cells, and the IL-1β (**H**), IL-18 (**I**) or IL-6 (**J**) concentration in the culture medium was determined by ELISA, and then normalized by protein concentration in cell lysates (n = 3). Data are presented as mean ± SEM of biologically independent samples. ∗ P < 0.05, ∗∗ P < 0.01. One-way ANOVA was used to analyze the data among multiple groups, followed by Tukey’s post hoc test

We next investigated whether DPP4 was involved in pyroptosis of HK-2 cells induced by HG. Silencing of DPP4 attenuated the HG-stimulated DPP4 expression and JNK phosphorylation and restored HG-suppressed SIRT3 and FGFR1 expression in HK-2 cells, indicating DPP4 upregulated JNK activation and suppressed SIRT3 and FGFR1 activity under HG conditions (Fig. 5A, B). The effect of DPP4 on HG-stimulated HK-2 cell pyroptosis was demonstrated by the reduced percentage of active Caspase 1 and PI labeled cells, as well as the attenuated intracellular Caspase 1 activity (Fig. 5C–E). Furthermore, knockdown of DPP4 decreased HG-augmented protein levels of NLRP3, cleaved-Caspase1, GSDMD-N, IL-1β, and IL-18 in HK-2 cells (Fig. 5F–G), accompanied by the blockage on HG-promoted release of IL-1β, IL-18 and IL-6 in HK-2 cells (Fig. 5H–J). Furthermore, DPP4 knockdown also alleviated the typical morphological changes of pyroptosis in HG-stimulated HK-2 cells (Additional file 1: Fig. S3). The above data suggested that DPP4 may facilitate HG-induced HK-2 cell pyroptosis by activating JNK.

### PRR exerted pyroptotic effects through DPP4-mediated signaling

**Fig. 6:**
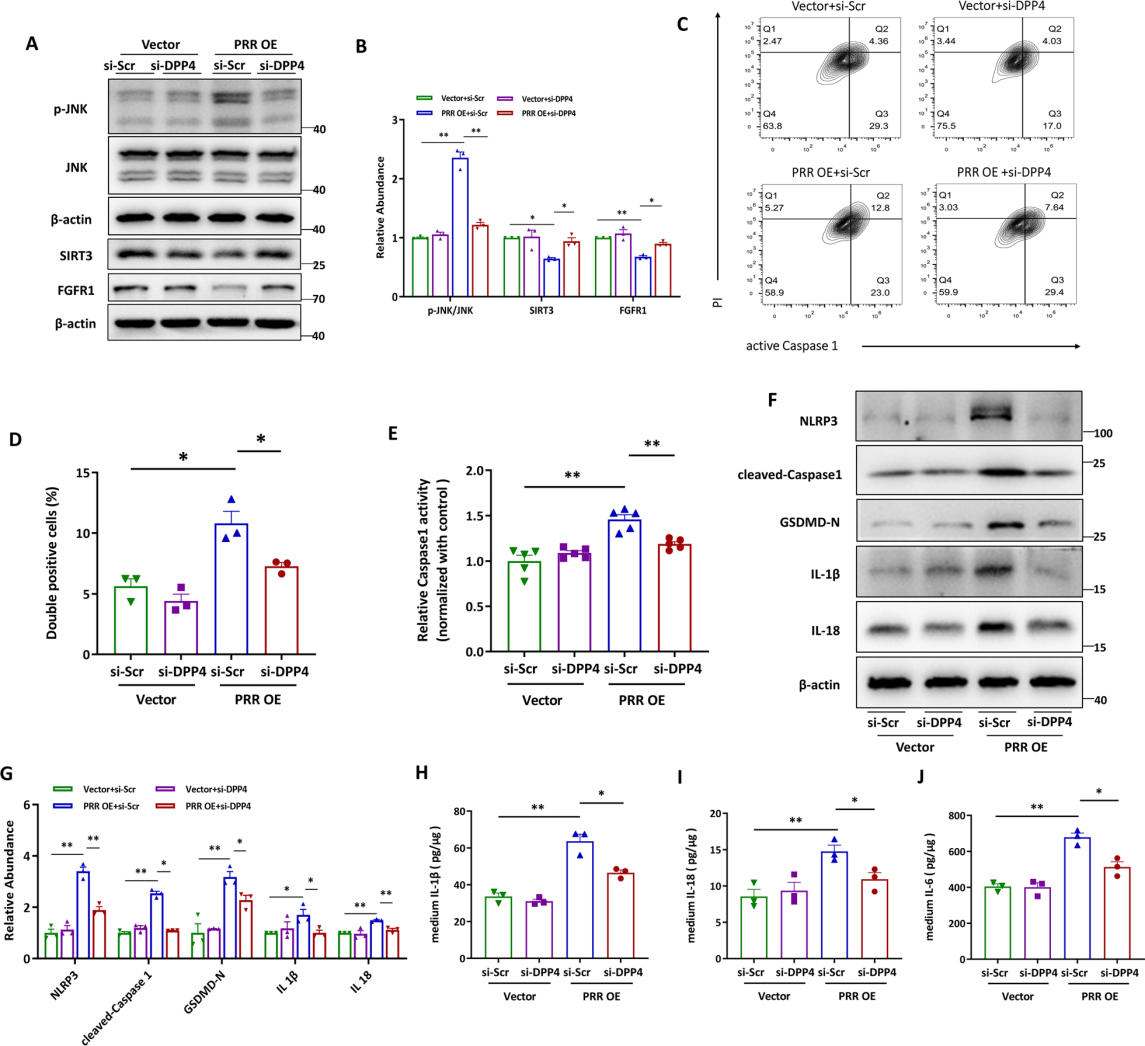
PRR exerted pyroptotic effects through DPP4-mediated signaling. **A** and **B** Representative western blot analyses (**A**) and quantitative data (**B**) showed that knocking down DPP4 by siRNA in HK-2 cells diminished PRR overexpression induced phosphorylation of JNK, and restored PRR overexpression reduced SIRT3 and FGFR1 expression (n = 3). **C** and **D** Flow cytometry analysis showed that knocking down DPP4 reduced PRR overexpression induced frequency of active Caspase1^+^PI^+^ HK-2 cells (**C**) and quantitative data (**D**) (n = 3). **E** PRR was silenced in HG stimulated HK-2 cells, and the Caspase1 activity in cell lysis was determined by kits. **f** and **g** Western blot analyses (**F**) and quantitative data (**G**) showed that DPP4 siRNA blocked HG-induced NLRP3, cleaved-Caspase1, GSDMD-N, IL-1β and IL-18 expression in HK-2 cells (n = 3). **H** and **I** DPP4 was knocked down in PRR overexpressed HK-2 cells, and the IL-1β (**H**), IL-18 (**I**) or IL-6 (**J**) concentration in the culture medium was determined by ELISA, and then normalized by protein concentration in cell lysates (n = 3). Data are presented as mean ± SEM of biologically independent samples. ∗ P < 0.05, ∗∗ P < 0.01. One-way ANOVA was used to analyze the data among multiple groups, followed by Tukey’s post hoc test

Next, we performed experiments to confirm whether PRR functioned through DPP4-regulated JNK activation, SIRT3 inhibition and FGFR1 suppression. Immunoblotting analyses showed that the promotional effects of PRR overexpression on JNK were blocked by DPP4 knockdown, indicating the influence of DPP4 on PRR-regulated JNK activation (Fig. 6A, B). Accordingly, PRR overexpression-induced HK-2 cell pyroptosis was also blunted by DPP4 silencing. DPP4 ablation reversed PRR overexpression-augmented increased percentage of active Caspase 1 and PI positive cells (Fig. 6C, D) and intracellular Caspase 1 activity (Fig. 6E). PRR overexpression promoted upregulation of NLRP3, cleaved-Caspase1, GSDMD-N, IL-1β, and IL-18 that was blunted by DPP4 knockdown (Fig. 6F, G). Moreover, decreased release of IL-1β, IL-18 and IL-6 in PRR-overexpressed HK-2 cells were also observed on DPP4 knockdown (Fig. 6H–J). The above data demonstrated that PRR exerted pyroptotic effects through DPP4-mediated JNK activation.

### Silencing of PRR ameliorated kidney injury and inflammation in *db/db* mice

**Fig. 7:**
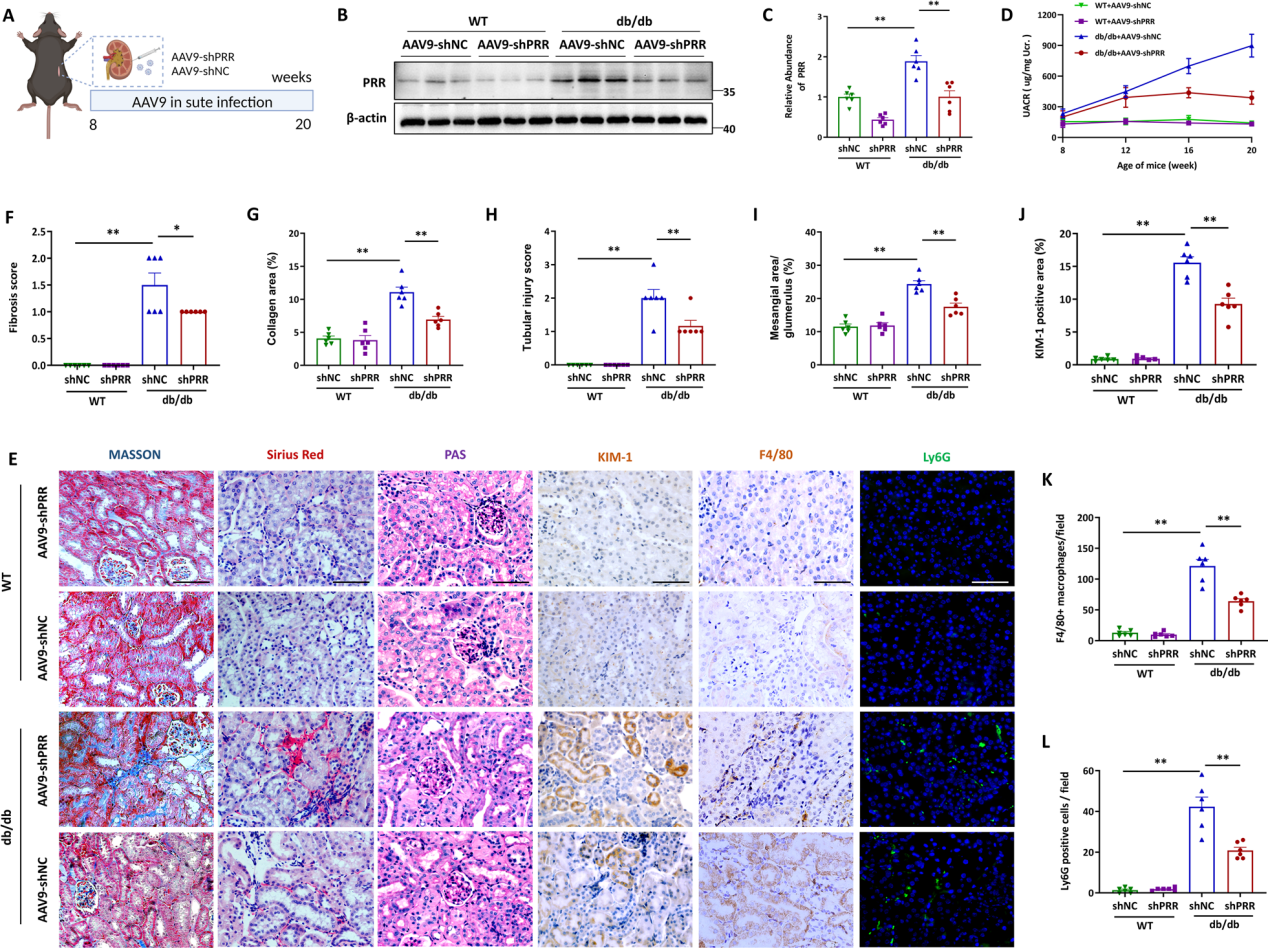
Silencing of PRR ameliorated kidney injury and inflammation in *db/db* mice. **A** Schematic diagram of the AAV injection surgery. **B** and **C** The protein level of PRR was assessed by Western blot. Representative Western blot (**B**) and quantitative data (**C**) are showed (n = 6). **D** Measured of urinary albumin concentration by using albumin detection kit, the quantitative data was normalized with urinary creatinine. **E**–**L** Representative images of Masson, Sirius Red, PAS, KIM-1, F4/80 and Ly6G staining of renal section of WT mice and *db/db* mice with or without PRR silence (**E**) and quantitative analysis (**F**–**L**) (n = 6). Bar = 20 μm. Data are presented as mean ± SEM of biologically independent samples. ∗ P < 0.05, ∗∗ P < 0.01. One-way ANOVA was used to analyze the data among multiple groups, followed by Tukey’s post hoc test

To further investigate the role of PRR in DKD, we infected the mice with adeno-associated virus (AAV) containing the interference sequence of PRR (AAV9-shPRR) by in situ injection into kidney. Then, the AAV-shPRR or control virus was injected to WT and *db/db* mice at the age of 8 weeks and the efficiency of PRR silencing in the kidneys was verified 3 months later (Fig. 7A). As shown, AAV-shPRR injection significantly decreased the renal PRR abundance (Fig. 7B, C). Furthermore, our results revealed that silencing of PRR significantly blunted the diabetic kidney injury in the *db/db* mice, compared with the untreated mice, as suggested by the reduced renal function loss from urinary albumin-to-creatinine ratio (UACR) assay (Fig. 7D) and the reduction of histological injury as assessed by Masson, Sirius Red, PAS and KIM-1 staining (Fig. 7E–J). Moreover, immunostaining showed that PRR knockdown blocked renal inflammation in the AAV9-shPRR treated *db/db* mice, as reflected by marked reduction of F4/80^+^ macrophage infiltration and Ly6G^+^ neutrophil infiltration (Fig. 7K–L). Collectively, these data indicated that ablation of PRR relieved tubule damage, renal inflammation, and kidney injury in diabetic mice.

### PRR knockdown blunted pyroptotic cell death and fibrotic response in *db/db* mice via DPP4-mediated signaling

**Fig. 8:**
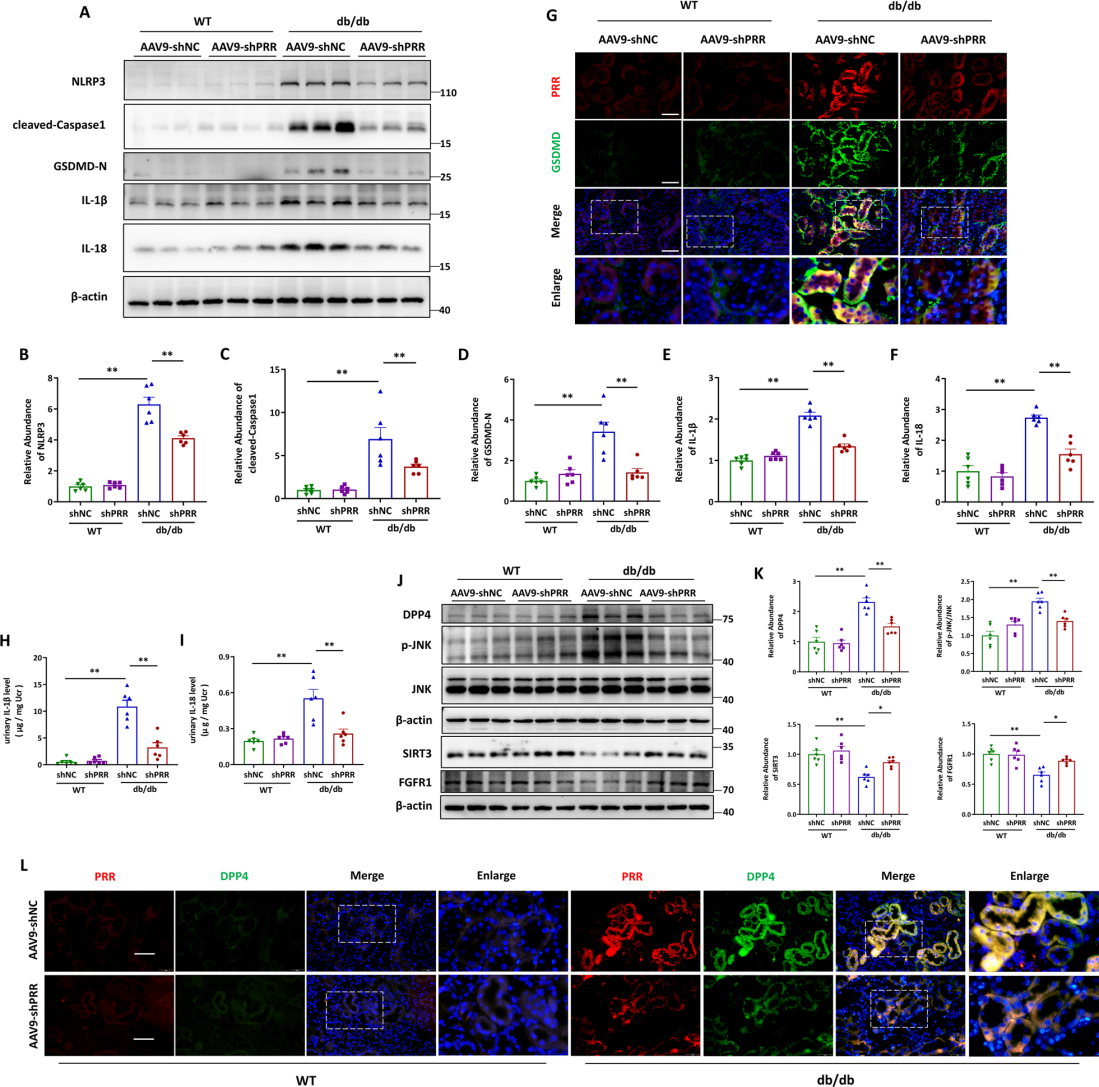
PRR knockdown blunted pyroptotic cell death and fibrotic response in *db/db* mice via DPP4-mediated signaling. **A**–**F** The protein levels of NLRP3, cleaved-Caspase1, GSDMD-N, IL-1β, IL-18 were assessed by Western blot. Representative Western blot (**A**) and quantitative data (**B**–**F**) are showed (n = 6). **C** Representative immunofluorescence microscopy images of coimmunostaining PRR (red) with GSDMD (green) in renal sections from WT and *db/db* mice with or without PRR ablation. Bar = 20 μm. **H** and **I** Elisa detection of urinary IL-1β (**H**) and IL-18 (**I**) concentration, the quantitative data were normalized with urinary creatine (n = 6). **J**–**K** western blot analyses (**J**) and quantitative data (**K**) showed the abundance of DPP4, phosphorylation level of JNK, SIRT3 and FGFR1 (n = 6). **L** Representative fluorescent images of renal sections from mice with or without PRR silence and stained with PRR (red), DPP4 (green). Bar = 20 μm. Data are presented as mean ± SEM of biologically independent samples. ∗ P < 0.05, ∗∗ P < 0.01. One-way ANOVA was used to analyze the data among multiple groups, followed by Tukey’s post hoc test

We next examined whether PRR ablation affected tubular cell pyroptosis and its effect on DPP4 mediated signaling. Western blot analyses showed that PRR knockdown reduced the protein level of NLRP3, cleaved-Caspase1, GSDMD-N, IL-1β, and IL18 in kidneys (Fig. 8A–F). Similar trends and the co-localization of PRR and GSDMD in renal tubules were observed by immunostaining (Fig. 8G). Furthermore, the urinary excretion of IL-1β and IL-18 was also inhibited in AAV-shPRR infected *db/db* mice (Fig. 8H, I), suggesting that tubular cell pyroptosis was attenuated in diabetic mice. Besides, the abundance of DPP4, phosphorylated JNK, SIRT3 and FGFR1 were significantly reversed (Fig. 8J–K), along with the reduced expression and co-localization of PRR and DPP4 in renal tubules of AAV9-shPRR infected *db/db* mice (Fig. 8L). These results suggested that PRR promoted the progression of DKD by regulating tubular epithelial cell pyroptosis via interaction with DPP4 to mediate the activity of JNK signaling, SIRT3 signaling and FGFR1 signaling. Furthermore, as DPP4 is an important inducer of EMT and EndMT, we examined the regulation of PRR on the markers of epithelial-to-mesenchymal transition (EMT) and endothelial-to-mesenchymal transition (EndMT) in kidneys. Western blot analyses showed that AAV9-shPRR reduced the upregulation of α-SMA and Vimentin and restored the downregulation of E-cadherin and CD31 in the kidney of db/db mice. Accompanied with the suppressed expression of DPP4 in AAV9-shPRR treated db/db mice, the markers of EMT and EndMT were reduced, indicating the alleviation of renal EMT and EndMT (Additional file 1: Fig. S5A, B).

## Discussion

Diabetic kidney disease is a serious complication of diabetes and a major cause of ESRD. However, effective therapies are limited due to the complexity of the pathogenic mechanism [1, 14]. It is well documented that renal inflammatory response is activated in both patients with DKD and experimental models [56–61]. Increasingly, studies have reported that PRR promotes inflammatory response that underlies the pathophysiological processes in a variety of tissues and organs, including kidney, liver, and heart [32, 62, 63]. However, the underlying mechanism remains unclear. In this study, we evaluated the expression of PRR in renal lesions of both diabetic mice and DKD patients and analyzed the effect of PRR on tubular epithelial cell (TEC) pyroptosis, which aggravates the progression of DKD. We found that tubular PRR and pyroptotic cell death of TECs were significantly elevated in the kidney biopsies of DKD patients. Knocking down PRR blocked renal damage in diabetic mice, as well as the pyroptotic cell death of TECs. Furthermore, we demonstrated that PRR interacted with DPP4 by binding with the cysteine-enrich region of C-terminal of DPP4 and stabilizing its protein abundance to activate JNK signaling and suppressing SIRT3 signaling and FGFR1 signaling, and then mediating the TEC pyroptosis.

PRR is a novel component of local RAS and promotes pathogenesis of various diseases in RAS-dependent or -independent manner, through regulation of renal control of electrolyte balance and blood pressure via activating local RAS, kidney fibrosis via interacting with β-catenin signaling, and hepatosteatosis by integrating hepatic lipid and glucose metabolism [29–31, 64]. Increasing evidence suggests the promotion by PRR of inflammatory response in different diseases, as reflected by induction of cytokine expression and immune cell infiltration in tissues by PRR inhibition; however, the underlying mechanisms of this effect remain unknown [32, 62, 63]. In this study, we found that the induced renal tubular expression of PRR in DKD patients was positively correlated with the clinical indexes, accompanied by the co-localization of increased GSDMD in tubules and the upregulation of urinary cytokine, indicating the pathogenic role of PRR in DKD and regulation of TECs pyroptosis. This hypothesis was further supported by the following experimental findings: knocking-down of PRR ameliorated kidney injury, renal inflammation, and TECs pyroptosis in *db/db* mice, as well as the HG-induced HK-2 cells pyroptosis. Meanwhile, in cultured HK-2 cells, overexpression of PRR promoted cell pyroptosis. These results revealed that PRR regulated the progression of DKD by promoting TECs pyroptosis. Previously, PRR was reported to be engaged in streptozotocin-induced type 1 diabetic kidney injury through its blocker handle region decoy peptide (HRP) [65–69]. However, the HRP used in previous studies was demonstrated as failing to prevent PRR signaling [70]. In the present study, we used adeno-associated virus vectors for silencing PRR in kidney to intuitively evaluate the role of PRR in type 2 DKD in *db/db* mice. More importantly, to our knowledge, this is the first study that demonstrated the role of PRR in pyroptotic cell death, which explained the regulatory role of PRR on inflammatory response in a variety of diseases.

In this study, we observed that high glucose level not only induced the expression of PRR, but also unexpectedly enhanced the physical binding of PRR with DPP4. To explore the mechanisms mediating PRR-promoted TEC pyroptosis, we analyzed the BioGrid database and found that DPP4 was a potential binding partner of PRR. The predicted interaction of PRR and DPP4 was verified by Co-IP analysis, which showed that endogenous PRR could bind DPP4 in HK-2 cells. Meanwhile, the co-localization of PRR and DPP4 was also observed in renal tubules of DKD patients and diabetic mice. To determine which domain of DPP4 interacted with PRR, DPP4-N-terminal (amino acids 1–324), DPP4-C-terminal (amino acids 325–766) and DPP4-C-terminal (cysteine-enrich domain, amino acids 325–551) plasmids were constructed and transfected into HK-2 cells. IP analyses showed the C-terminus of DPP4 binding with PRR, indicating that PRR interacted with DPP4 by binding to the cysteine-enrich region in C-terminal of DPP4. Furthermore, the protein level of DPP4 was also regulated by PRR both in vitro and in vivo. Meanwhile, the effect of DPP4 on HG or PRR overexpression-induced HK-2 cell pyroptosis was demonstrated by silencing DPP4. Mitogen-activated protein kinases (JNK, ERK, and p38) serve as central stress and immune integration hubs during inflammatory responses and mediate the signal transduction of PRR [71–73]. A recent study demonstrated that JNK facilitated pyroptotic signaling that was required for dextran sulfate sodium-induced colitis in mice [74]. Meanwhile, SIRT3 and FGFR1 are important molecules in regulating pytopyotic cell death and kidney fibrosis. Hence, we detected the involvement of JNK signaling, SIRT3 signaling and FGFR1 signaling in PRR-promoted pyroptotic cell death, and found that ablation of PRR or DPP4 blocked HG or PRR overexpression-regulated JNK activation, SIRT3 suppression and FGFR1 inhibition. Taken together, we demonstrated that PRR promoted TEC pyroptosis via interaction with DPP4 to activate JNK signaling, and to block SIRT3 signaling and FGFR1 signaling.

Our study, however, had some limitations. For example, to investigate the role of PRR in DKD, transgenic or knockout mice could provide more convincing evidence, although AAV9 showed high efficiency in mediating gene transfer. Because we observed significant increase of PRR in renal tubules, which co-localized with upregulated GSDMD, tubular-specific deletion or overexpression of PRR would be a better strategy to evaluate the involvement of PRR in DKD. Nonetheless, to our knowledge, our study demonstrated for the first time that PRR induced tubular epithelial cell pyroptosis to aggravate DKD. Besides, a small number of human kidney biopsy specimens was used in the current study, and multivariate analysis was not conducted for this reason. The positive association between tubular PRR abundance and kidney injury index of DKD patients should be confirmed by larger studies with numerous human kidney biopsy samples from different regions. Furthermore, the role of local renin-angiotensin system (RAS) in PRR regulated pyroptotic cell death in DKD needs further exploration. Our results showed that PRR could promote HK-2 pyroptosis independent of angiotensin II (Ang II) in vitro, but not fully exclude the role of Ang II signaling in the mediation of PRR regulated pyroptosis in DKD in vivo. The animal experiment of losartan treatment on renal PRR overexpressed *db/db* mice should be conducted in the followed study to detect the engagement of local RAS in PRR regulated pytoptotic cell death in DKD. Moreover, the exact molecular mechanisms by which PRR modulates DPP4 protein expression remain unclear. In this study, we demonstrated that ablation or overexpression of PRR in HK-2 cells regulated DPP4 protein abundance, but the underlying mechanism needs further investigation. A previous study provided evidence showing that DPP4 interacts with USP26 and protected from ubiquitin-mediated degradation in human aortic valve interstitial cells [75]. However, whether PRR-mediated DPP4 stabilization is also deubiquitination-dependent needs to be elucidated.

## Conclusions

The present study intuitively identified the pathogenic role of PRR in DKD. PRR via binding with the cysteine-enrich region in C-terminal of DPP4 to regulate the activity of JNK, SIRT3 and FGFR1 to promote pyroptotic cell death of TECs, and then aggravated the progression of DKD. Overall, these results helped define PRR as a potential therapeutic target in DKD.

### Supplementary Information

**Additional file 1: Figure S1. **Schematic diagram showing the injection sites of adeno-associated virus (AAV9-shPRR or negative control (AAV9-shNC). **Figure S2. **Pathological changes of glomerulus in DKD patients. **A**, **B** Representative transmission electron microscopy images of glomerular basement membrane in renal sections from DKD patients and health controls (**A**) and quantitative analysis of glomerular basement membrane (GBM) (**B**). n=5. Bar=1 μm **C**–**F** Representative immunohistochemistry staining images of WT-1 and CD31 in renal sections from DKD patients and health controls (**C**, **E**) and quantitative analysis (**D**, **F**). n=5. Bar=10μm. Data are presented as mean ± SEM of biologically independent samples. ∗∗P < 0.01. *P* values were determined by Student’s t-test for comparison between two groups. **Figure S3. **Knockdown of PRR or DPP4 alleviated high glucose induced pyroptotic morphological changes of HK-2 cells. Representative scanning electron microscope (SEM) images of HK-2 cells under different treatments. Scale bars: 20 μm. **Figure S4. **PRR exerted pyroptotic effects independent of Ang II in HK-2 cells. **A** and **B** Representative western blot analyses (**A**) and quantitative data (**B**) showed that blocking Ang II receptor with losartan had no significant effect on PRR overexpression induced protein expression of NLRP3, cleaved-Caspase1, GSDMD-N, IL-1β and IL-18 in HK-2 cells (n = 3). Data are presented as mean ± SEM of biologically independent samples. ∗P < 0.05, ∗∗P < 0.01, ns P>0.05. One-way ANOVA was used to analyze the data among multiple groups, followed by Tukey’s post hoc test. **Figure S5. **PRR promoted EMT and EndMT in the kidney of db/db mice. **A** and **B** Representative western blot analyses (**A**) and quantitative data (**B**) showed that AAV9-shPRR reduced the expression of α-SMA and Vimentin and restored the abundance of E-Cadherin and CD 31in the kidney of db/db mice (n = 6). Data are presented as mean ± SEM of biologically independent samples. ∗∗P < 0.01. One-way ANOVA was used to analyze the data among multiple groups, followed by Tukey’s post hoc test.

**Additional file 2: Table S1.** Parameter values of DKD patients. **Table S2.** Parameter values of healthy subjects and DKD patients.

## Data Availability

The dataset used and analyzed in our study are available from the GEO database (https://www.ncbi.nlm.nih.gov/geo/). All data presented in this study are included within the article (and its additional files). The additional data supporting the findings of this study could be obtained from the corresponding author upon reasonable request.

## Abbreviations

PRR: (Pro)renin receptor
DPP4: Dipeptidyl peptidase 4
DKD: Diabetic kidney disease
AAV9: Adeno-associated viral 9
TECs: Tubular epithelial cells
HK-2: Human renal tubular epithelial cell line
JNK: C-Jun N-terminal kinase
ERSD: End-stage renal disease
NLRP3: NOD-, LRR-, and pyrin-domain containing protein 3
RAS: Renin-angiotensin system
PAS: Periodic Acid-Schiff
UACR: Urine albumin/creatinine ratio
IL-1β: Interleukin-1β
IL-18: Interleukin-18
eGFR: Estimated glomerular filtration rate
BUN: Blood urea nitrogen
HRP: Handle region decoy peptide

## References

[1] Doshi SM, Friedman AN (2017). Diagnosis and management of type 2 diabetic kidney disease. Clin J Am Soc Nephrol.

[2] Broz P, Pelegrin P, Shao F (2020). The gasdermins, a protein family executing cell death and inflammation. Nat Rev Immunol.

[3] Li J, Liu H, Takagi S, Nitta K, Kitada M, Srivastava SP (2020). Renal protective effects of empagliflozin via inhibition of EMT and aberrant glycolysis in proximal tubules. JCI Insight.

[4] Li J, Shi S, Srivastava SP, Kitada M, Nagai T, Nitta K (2017). FGFR1 is critical for the anti-endothelial mesenchymal transition effect of N-acetyl-seryl-aspartyl-lysyl-proline via induction of the MAP4K4 pathway. Cell Death Dis.

[5] Shi S, Srivastava SP, Kanasaki M, He J, Kitada M, Nagai T (2015). Interactions of DPP-4 and integrin beta1 influences endothelial-to-mesenchymal transition. Kidney Int.

[6] Srivastava SP, Goodwin JE, Kanasaki K, Koya D (2020). Metabolic reprogramming by N-acetyl-seryl-aspartyl-lysyl-proline protects against diabetic kidney disease. Br J Pharmacol.

[7] Srivastava SP, Goodwin JE, Kanasaki K, Koya D (2020). Inhibition of angiotensin-converting enzyme ameliorates renal fibrosis by mitigating DPP-4 level and restoring antifibrotic MicroRNAs. Genes.

[8] Srivastava SP, Li J, Kitada M, Fujita H, Yamada Y, Goodwin JE (2018). SIRT3 deficiency leads to induction of abnormal glycolysis in diabetic kidney with fibrosis. Cell Death Dis.

[9] Srivastava SP, Li J, Takagaki Y, Kitada M, Goodwin JE, Kanasaki K (2021). Endothelial SIRT3 regulates myofibroblast metabolic shifts in diabetic kidneys. iScience.

[10] Liu H, Takagaki Y, Kumagai A, Kanasaki K, Koya D (2021). The PKM2 activator TEPP-46 suppresses kidney fibrosis via inhibition of the EMT program and aberrant glycolysis associated with suppression of HIF-1alpha accumulation. J Diabetes Investig.

[11] Guo B, Inoki K, Isono M, Mori H, Kanasaki K, Sugimoto T (2005). MAPK/AP-1-dependent regulation of PAI-1 gene expression by TGF-beta in rat mesangial cells. Kidney Int.

[12] Srivastava SP, Zhou H, Setia O, Liu B, Kanasaki K, Koya D (2021). Loss of endothelial glucocorticoid receptor accelerates diabetic nephropathy. Nat Commun.

[13] Zhao XP, Chang SY, Pang Y, Liao MC, Peng J, Ingelfinger JR (2023). Hedgehog interacting protein activates sodium-glucose cotransporter 2 expression and promotes renal tubular epithelial cell senescence in a mouse model of type 1 diabetes. Diabetologia.

[14] Tuttle KR, Agarwal R, Alpers CE, Bakris GL, Brosius FC, Kolkhof P (2022). Molecular mechanisms and therapeutic targets for diabetic kidney disease. Kidney Int.

[15] Liu S, Yuan Y, Xue Y, Xing C, Zhang B (2022). Podocyte injury in diabetic kidney disease: a focus on mitochondrial dysfunction. Front Cell Dev Biol.

[16] Bonventre JV (2012). Can we target tubular damage to prevent renal function decline in diabetes?. Semin Nephrol.

[17] Gilbert RE (2017). Proximal tubulopathy: prime mover and key therapeutic target in diabetic kidney disease. Diabetes.

[18] Qu X, Zhai B, Liu Y, Chen Y, Xie Z, Wang Q (2022). Pyrroloquinoline quinone ameliorates renal fibrosis in diabetic nephropathy by inhibiting the pyroptosis pathway in C57BL/6 mice and human kidney 2 cells. Biomed Pharmacother.

[19] Coll RC, Schroder K, Pelegrin P (2022). NLRP3 and pyroptosis blockers for treating inflammatory diseases. Trends Pharmacol Sci.

[20] Li Y, Yuan Y, Huang ZX, Chen H, Lan R, Wang Z (2021). GSDME-mediated pyroptosis promotes inflammation and fibrosis in obstructive nephropathy. Cell Death Differ.

[21] Zhang Z, Zhang Y, Xia S, Kong Q, Li S, Liu X (2020). Gasdermin E suppresses tumour growth by activating anti-tumour immunity. Nature.

[22] Zheng F, Ma L, Li X, Wang Z, Gao R, Peng C (2022). Neutrophil extracellular traps induce glomerular endothelial cell dysfunction and pyroptosis in diabetic kidney disease. Diabetes.

[23] Baatarjav C, Komada T, Karasawa T, Yamada N, Sampilvanjil A, Matsumura T (2022). dsDNA-induced AIM2 pyroptosis halts aberrant inflammation during rhabdomyolysis-induced acute kidney injury. Cell Death Differ.

[24] Pang Q, Wang P, Pan Y, Dong X, Zhou T, Song X (2022). Irisin protects against vascular calcification by activating autophagy and inhibiting NLRP3-mediated vascular smooth muscle cell pyroptosis in chronic kidney disease. Cell Death Dis.

[25] Lan J, Xu B, Shi X, Pan Q, Tao Q (2022). WTAP-mediated N(6)-methyladenosine modification of NLRP3 mRNA in kidney injury of diabetic nephropathy. Cell Mol Biol Lett.

[26] Malek V, Suryavanshi SV, Sharma N, Kulkarni YA, Mulay SR, Gaikwad AB (2021). Potential of renin-angiotensin-aldosterone system modulations in diabetic kidney disease: old players to new hope!. Rev Physiol Biochem Pharmacol.

[27] Thomas MC, Brownlee M, Susztak K, Sharma K, Jandeleit-Dahm KA, Zoungas S (2015). Diabetic kidney disease. Nat Rev Dis Primers.

[28] Ichihara A, Yatabe MS (2019). The (pro)renin receptor in health and disease. Nat Rev Nephrol.

[29] Ren L, Sun Y, Lu H, Ye D, Han L, Wang N (2018). (Pro)renin receptor inhibition reprograms hepatic lipid metabolism and protects mice from diet-induced obesity and hepatosteatosis. Circ Res.

[30] Fu Z, Hu J, Zhou L, Chen Y, Deng M, Liu X (2019). (Pro)renin receptor contributes to pregnancy-induced sodium-water retention in rats via activation of intrarenal RAAS and alpha-ENaC. Am J Physiol Renal Physiol.

[31] Li Z, Zhou L, Wang Y, Miao J, Hong X, Hou FF (2017). (Pro)renin receptor is an amplifier of Wnt/beta-catenin signaling in kidney injury and fibrosis. J Am Soc Nephrol.

[32] Fang H, Deng M, Zhang L, Lu A, Su J, Xu C (2018). Role of (pro)renin receptor in albumin overload-induced nephropathy in rats. Am J Physiol Renal Physiol.

[33] Ma H, Dong XF, Cao XR, Hei NH, Li JL, Wang YL (2020). Pro-renin receptor overexpression promotes angiotensin II-induced abdominal aortic aneurysm formation in apolipoprotein E-knockout mice. Hum Gene Ther.

[34] Kanda A, Ishizuka ET, Shibata A, Matsumoto T, Toyofuku H, Noda K (2017). A novel single-strand RNAi therapeutic agent targeting the (Pro)renin receptor suppresses ocular inflammation. Mol Ther Nucleic Acids.

[35] Nistala R, Savin V (2017). Diabetes, hypertension, and chronic kidney disease progression: role of DPP4. Am J Physiol Renal Physiol.

[36] Deacon CF (2019). Physiology and pharmacology of DPP-4 in glucose homeostasis and the treatment of type 2 diabetes. Front Endocrinol.

[37] Wang N, Shi X, Jiang L, Zhang S, Wang D, Tong P (2013). Structure of MERS-CoV spike receptor-binding domain complexed with human receptor DPP4. Cell Res.

[38] Alicic RZ, Cox EJ, Neumiller JJ, Tuttle KR (2021). Incretin drugs in diabetic kidney disease: biological mechanisms and clinical evidence. Nat Rev Nephrol.

[39] Iwakura T, Zhao Z, Marschner JA, Devarapu SK, Yasuda H, Anders HJ (2019). Dipeptidyl peptidase-4 inhibitor teneligliptin accelerates recovery from cisplatin-induced acute kidney injury by attenuating inflammation and promoting tubular regeneration. Nephrol Dial Transplant.

[40] Tang PM, Zhang YY, Hung JS, Chung JY, Huang XR, To KF (2021). DPP4/CD32b/NF-kappaB circuit: a novel druggable target for inhibiting CRP-driven diabetic nephropathy. Mol Ther.

[41] Li J, Liu H, Srivastava SP, Hu Q, Gao R, Li S (2020). Endothelial FGFR1 (Fibroblast Growth Factor Receptor 1) deficiency contributes differential fibrogenic effects in kidney and heart of diabetic mice. Hypertension.

[42] Gao R, Kanasaki K, Li J, Kitada M, Okazaki T, Koya D (2019). betaklotho is essential for the anti-endothelial mesenchymal transition effects of N-acetyl-seryl-aspartyl-lysyl-proline. FEBS Open Bio.

[43] Yang F, Takagaki Y, Yoshitomi Y, Ikeda T, Li J, Kitada M (2019). Inhibition of dipeptidyl peptidase-4 accelerates epithelial-mesenchymal transition and breast cancer metastasis via the CXCL12/CXCR4/mTOR Axis. Cancer Res.

[44] Kanasaki K, Shi S, Kanasaki M, He J, Nagai T, Nakamura Y (2014). Linagliptin-mediated DPP-4 inhibition ameliorates kidney fibrosis in streptozotocin-induced diabetic mice by inhibiting endothelial-to-mesenchymal transition in a therapeutic regimen. Diabetes.

[45] Shi S, Kanasaki K, Koya D (2016). Linagliptin but not Sitagliptin inhibited transforming growth factor-beta2-induced endothelial DPP-4 activity and the endothelial-mesenchymal transition. Biochem Biophys Res Commun.

[46] America Diabetes Association (2010). Standards of medical care in diabetes–2010. Diabetes Care.

[47] Tervaert TWC, Mooyaart AL, Amann K, Cohen AH, Cook HT, Drachenberg CB (2010). Pathologic classification of diabetic nephropathy. J Am Soc Nephrol.

[48] Woroniecka KI, Park AS, Mohtat D, Thomas DB, Pullman JM, Susztak K (2011). Transcriptome analysis of human diabetic kidney disease. Diabetes.

[49] Li J, Niu J, Min W, Ai J, Lin X, Miao J (2022). B7–1 mediates podocyte injury and glomerulosclerosis through communication with Hsp90ab1-LRP5-beta-catenin pathway. Cell Death Differ.

[50] Liu S, Li X, Wen R, Chen L, Yang Q, Song S (2022). Increased thromboxane/prostaglandin receptors contribute to high glucose-induced podocyte injury and mitochondrial fission through ROCK1-Drp1 signaling. Int J Biochem Cell Biol.

[51] Deng Q, Wen R, Liu S, Chen X, Song S, Li X (2020). Increased long noncoding RNA maternally expressed gene 3 contributes to podocyte injury induced by high glucose through regulation of mitochondrial fission. Cell Death Dis.

[52] Ma M, Li H, Yin S, Lin T, Song T (2023). Overexpression of miR-92a attenuates kidney ischemia-reperfusion injury and improves kidney preservation by inhibiting MEK4/JNK1-related autophagy. Cell Mol Biol Lett.

[53] Xie S, Su J, Lu A, Lai Y, Mo S, Pu M (2020). Soluble (pro)renin receptor promotes the fibrotic response in renal proximal tubule epithelial cells in vitro via the Akt/beta-catenin/Snail signaling pathway. Am J Physiol Renal Physiol.

[54] Yu P, Zhang X, Liu N, Tang L, Peng C, Chen X (2021). Pyroptosis: mechanisms and diseases. Signal Transduct Target Ther.

[55] Oughtred R, Rust J, Chang C, Breitkreutz BJ, Stark C, Willems A (2021). The BioGRID database: a comprehensive biomedical resource of curated protein, genetic, and chemical interactions. Protein Sci.

[56] Tang SCW, Yiu WH (2020). Innate immunity in diabetic kidney disease. Nat Rev Nephrol.

[57] Fu J, Sun Z, Wang X, Zhang T, Yuan W, Salem F (2022). The single-cell landscape of kidney immune cells reveals transcriptional heterogeneity in early diabetic kidney disease. Kidney Int.

[58] Rayego-Mateos S, Rodrigues-Diez RR, Fernandez-Fernandez B, Mora-Fernandez C, Marchant V, Donate-Correa J (2023). Targeting inflammation to treat diabetic kidney disease: the road to 2030. Kidney Int.

[59] Wang F, Hou W, Li X, Lu L, Huang T, Zhu M (2022). SETD8 cooperates with MZF1 to participate in hyperglycemia-induced endothelial inflammation via elevation of WNT5A levels in diabetic nephropathy. Cell Mol Biol Lett.

[60] Zhao M, Wang S, Zuo A, Zhang J, Wen W, Jiang W (2021). HIF-1alpha/JMJD1A signaling regulates inflammation and oxidative stress following hyperglycemia and hypoxia-induced vascular cell injury. Cell Mol Biol Lett.

[61] Sepehri Z, Kiani Z, Nasiri AA, Kohan F (2016). Toll-like receptor 2 and type 2 diabetes. Cell Mol Biol Lett.

[62] Hsieh YC, Lee KC, Lei HJ, Lan KH, Huo TI, Lin YT (2021). (Pro)renin receptor knockdown attenuates liver fibrosis through inactivation of ERK/TGF-beta1/SMAD3 pathway. Cell Mol Gastroenterol Hepatol.

[63] Yoshida A, Kanamori H, Naruse G, Minatoguchi S, Iwasa M, Yamada Y (2019). (Pro)renin receptor blockade ameliorates heart failure caused by chronic kidney disease. J Card Fail.

[64] Chavez-Canales M, Gamba G (2021). (Pro)renin receptor deletion in distal convoluted tubule 1 produces salt-sensitive hypertension. Hypertension.

[65] Matavelli LC, Huang J, Siragy HM (2010). (Pro)renin receptor contributes to diabetic nephropathy by enhancing renal inflammation. Clin Exp Pharmacol Physiol.

[66] Kokeny G, Fang L, Revesz C, Mozes MM, Voros P, Szenasi G (2017). The effect of combined treatment with the (Pro)Renin receptor blocker HRP and quinapril in type 1 diabetic rats. Kidney Blood Press Res.

[67] te Riet L, van den Heuvel M, Peutz-Kootstra CJ, van Esch JH, van Veghel R, Garrelds IM (2014). Deterioration of kidney function by the (pro)renin receptor blocker handle region peptide in aliskiren-treated diabetic transgenic (mRen2)27 rats. Am J Physiol Renal Physiol.

[68] Takahashi H, Ichihara A, Kaneshiro Y, Inomata K, Sakoda M, Takemitsu T (2007). Regression of nephropathy developed in diabetes by (Pro)renin receptor blockade. J Am Soc Nephrol.

[69] Ichihara A, Suzuki F, Nakagawa T, Kaneshiro Y, Takemitsu T, Sakoda M (2006). Prorenin receptor blockade inhibits development of glomerulosclerosis in diabetic angiotensin II type 1a receptor-deficient mice. J Am Soc Nephrol.

[70] Feldt S, Maschke U, Dechend R, Luft FC, Muller DN (2008). The putative (pro)renin receptor blocker HRP fails to prevent (pro)renin signaling. J Am Soc Nephrol.

[71] Ren Q, Guo F, Tao S, Huang R, Ma L, Fu P (2020). Flavonoid fisetin alleviates kidney inflammation and apoptosis via inhibiting Src-mediated NF-kappaB p65 and MAPK signaling pathways in septic AKI mice. Biomed Pharmacother.

[72] Wang Y, He G, Tang H, Shi Y, Kang X, Lyu J (2019). Aspirin inhibits inflammation and scar formation in the injury tendon healing through regulating JNK/STAT-3 signalling pathway. Cell Prolif.

[73] Huang J, Siragy HM (2010). Regulation of (pro)renin receptor expression by glucose-induced mitogen-activated protein kinase, nuclear factor-kappaB, and activator protein-1 signaling pathways. Endocrinology.

[74] Bradfield CJ, Liang JJ, Ernst O, John SP, Sun J, Ganesan S (2023). Biphasic JNK signaling reveals distinct MAP3K complexes licensing inflammasome formation and pyroptosis. Cell Death Differ.

[75] Wang Y, Han D, Zhou T, Chen C, Cao H, Zhang JZ (2021). DUSP26 induces aortic valve calcification by antagonizing MDM2-mediated ubiquitination of DPP4 in human valvular interstitial cells. Eur Heart J.

