# Supplementary material for: (Pro)renin receptor mediates tubular epithelial cell pyroptosis in diabetic kidney disease via DPP4-JNK pathway
Source: J Transl Med. 2024 Jan 5;22:26. doi: 10.1186/s12967-023-04846-5 (PMC10768114; doi:10.1186/s12967-023-04846-5)
Supplement: Supplementary file 1 — Additional file 1: Figure S1. Schematic diagram showing the injection sites of adeno-associated virus (AAV9-shPRR or negative control (AAV9-shNC). Figure S2. Pathological changes of glomerulus in DKD patients. A, B Representative transmission electron microscopy images of glomerular basement membrane in renal sections from DKD patients and health controls (A) and quantitative analysis of glomerular basement membrane (GBM) (B). n=5. Bar=1 μm C–F Representative immunohistochemistry staining images of WT-1 and CD31 in renal sections from DKD patients and health controls (C, E) and quantitative analysis (D, F). n=5. Bar=10μm. Data are presented as mean ± SEM of biologically independent samples. ∗∗P < 0.01. P values were determined by Student’s t-test for comparison between two groups. Figure S3. Knockdown of PRR or DPP4 alleviated high glucose induced pyroptotic morphological changes of HK-2 cells. Representative scanning electron microscope (SEM) images of HK-2 cells under different treatments. Scale bars: 20 μm. Figure S4. PRR exerted pyroptotic effects independent of Ang II in HK-2 cells. A and B Representative western blot analyses (A) and quantitative data (B) showed that blocking Ang II receptor with losartan had no significant effect on PRR overexpression induced protein expression of NLRP3, cleaved-Caspase1, GSDMD-N, IL-1β and IL-18 in HK-2 cells (n = 3). Data are presented as mean ± SEM of biologically independent samples. ∗P < 0.05, ∗∗P < 0.01, ns P>0.05. One-way ANOVA was used to analyze the data among multiple groups, followed by Tukey’s post hoc test. Figure S5. PRR promoted EMT and EndMT in the kidney of db/db mice. A and B Representative western blot analyses (A) and quantitative data (B) showed that AAV9-shPRR reduced the expression of α-SMA and Vimentin and restored the abundance of E-Cadherin and CD 31in the kidney of db/db mice (n = 6). Data are presented as mean ± SEM of biologically independent samples. ∗∗P < 0.01. One-way ANOVA was used to analyze [file 12967_2023_4846_MOESM1_ESM.docx]

**Supplementary Figures and Legends**


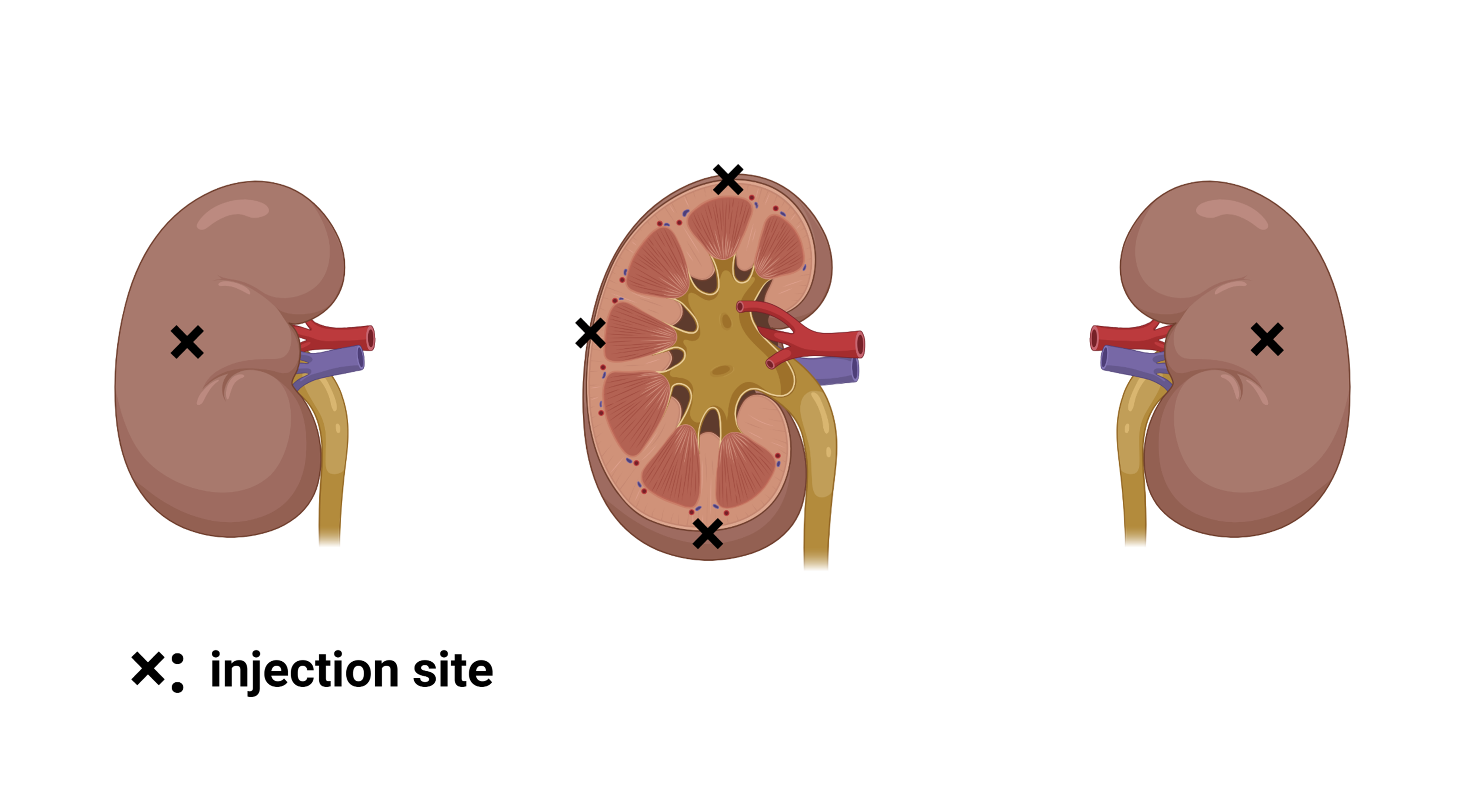


**Supplementary Figure 1**

Schematic diagram showing the injection sites of adeno-associated virus (AAV9-shPRR or negative control (AAV9-shNC).


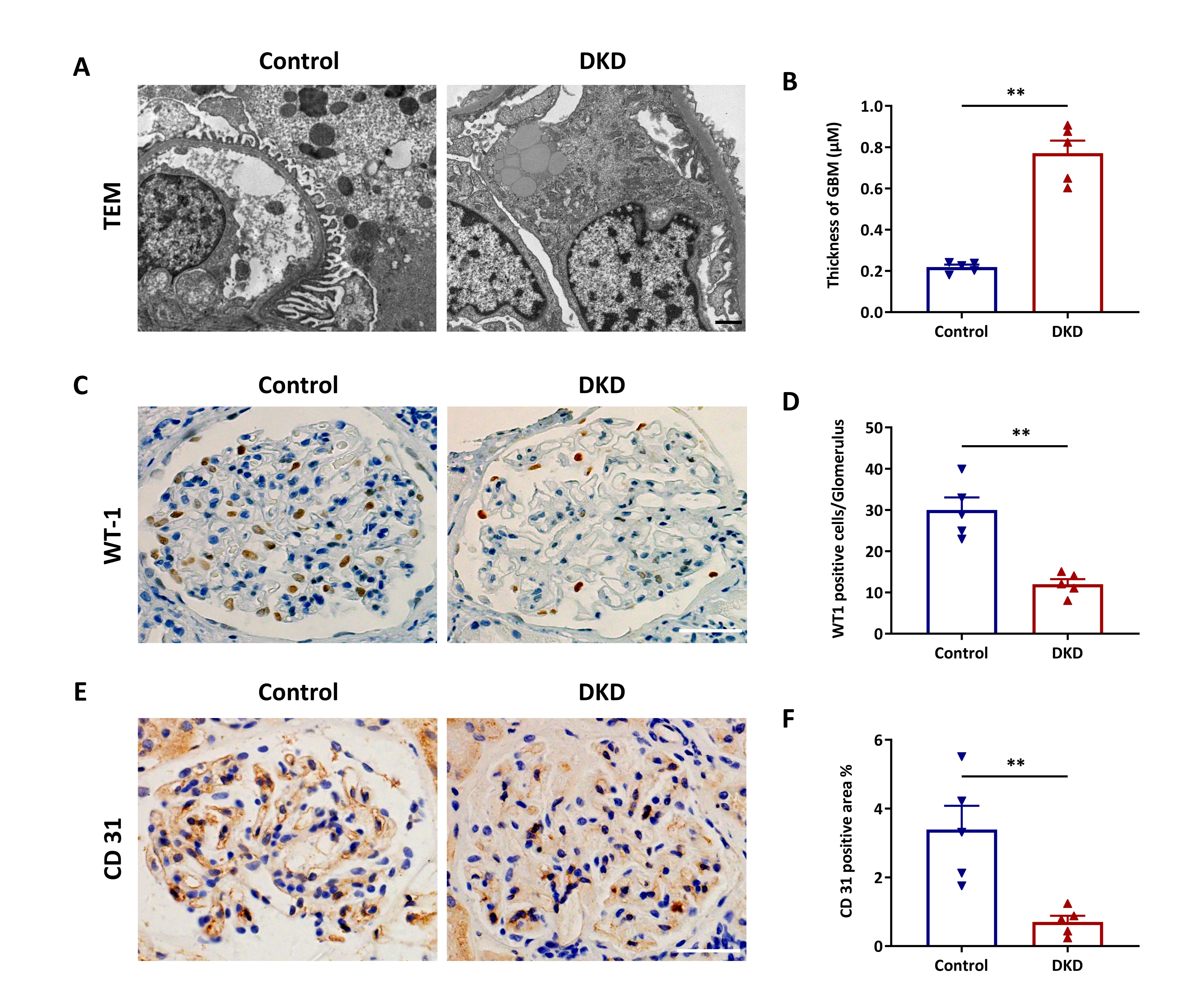


**Supplementary Figure 2**

Pathological changes of glomerulus in DKD patients. (A-B) Representative transmission electron microscopy images of glomerular basement membrane in renal sections from DKD patients and health controls (A) and quantitative analysis of glomerular basement membrane (GBM) (B). n=5. Bar=1μm. (C-F) Representative immunohistochemistry staining images of WT-1 and CD31 in renal sections from DKD patients and health controls (C, E) and quantitative analysis (D, F). n=5. Bar=10μm. Data are presented as mean ± SEM of biologically independent samples. ∗∗P < 0.01. *P* values were determined by Student's t-test for comparison between two groups.

**Supplementary Figure 3**

Knockdown of PRR or DPP4 alleviated high glucose induced pyroptotic morphological changes of HK-2 cells. Representative scanning electron microscope (SEM) images of HK-2 cells under different treatments. Scale bars: 20μm.


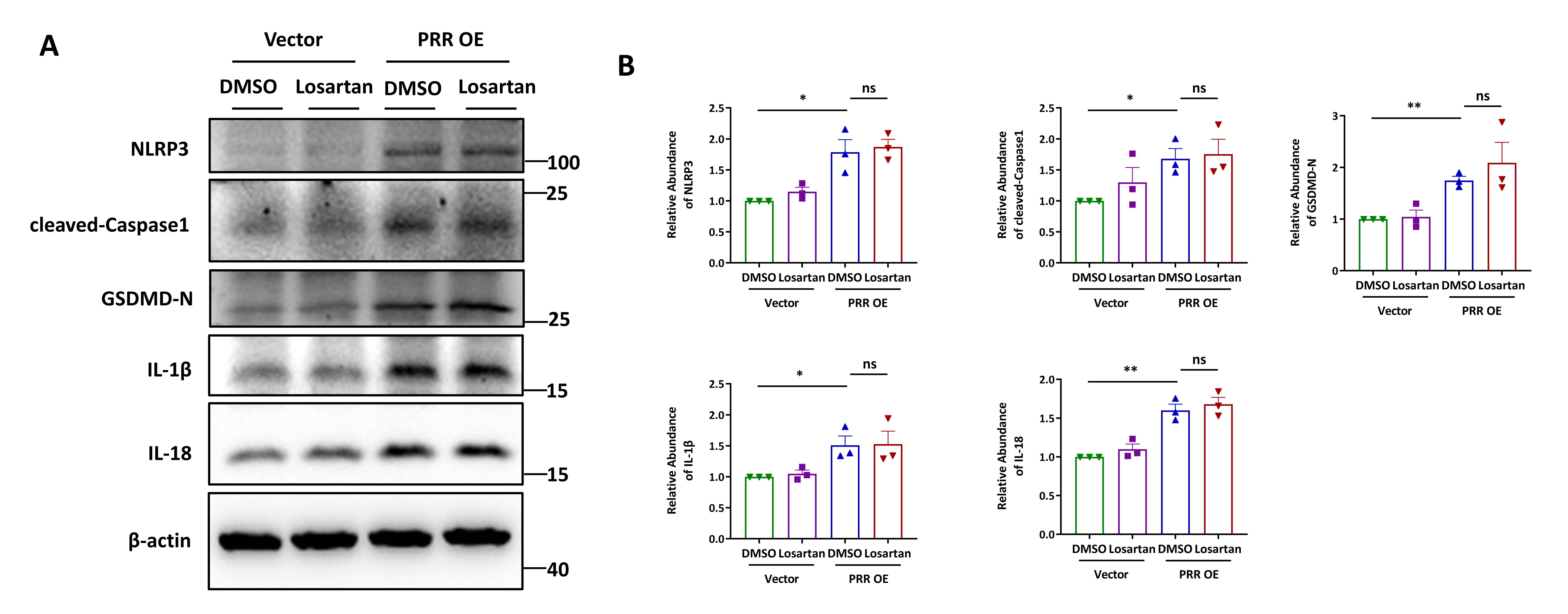


**Supplementary Figure 4**

PRR exerted pyroptotic effects independent of Ang II in HK-2 cells. (A and B) Representative western blot analyses (A) and quantitative data (B) showed that blocking Ang II receptor with losartan had no significant effect on PRR overexpression induced protein expression of NLRP3, cleaved-Caspase1, GSDMD-N, IL-1β and IL-18 in HK-2 cells (n = 3). Data are presented as mean ± SEM of biologically independent samples. ∗P < 0.05, ∗∗P < 0.01, ns P>0.05. One-way ANOVA was used to analyze the data among multiple groups, followed by Tukey’s post hoc test.


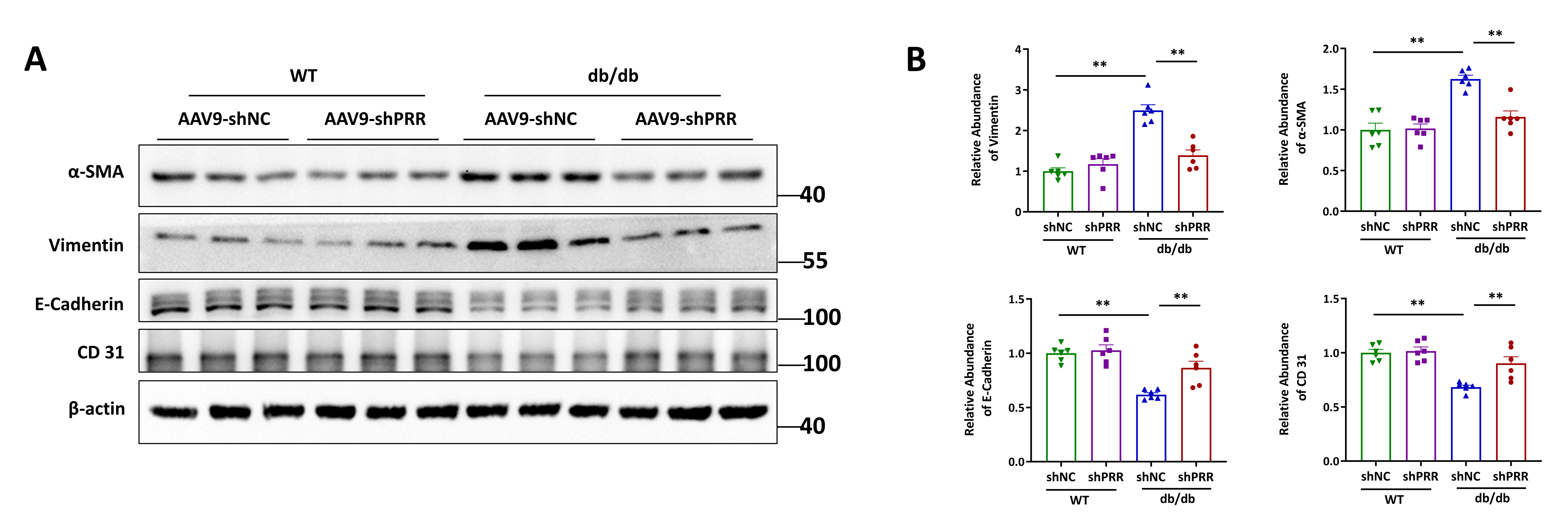


**Supplementary Figure 5**

PRR promoted EMT and EndMT in the kidney of db/db mice. (A and B) Representative western blot analyses (A) and quantitative data (B) showed that AAV9-shPRR reduced the expression of α-SMA and Vimentin and restored the abundance of E-Cadherin and CD 31in the kidney of db/db mice (n = 6). Data are presented as mean ± SEM of biologically independent samples. ∗∗P < 0.01. One-way ANOVA was used to analyze the data among multiple groups, followed by Tukey’s post hoc test.
