# Supplementary material for: (Pro)renin receptor mediates tubular epithelial cell pyroptosis in diabetic kidney disease via DPP4-JNK pathway
Source: J Transl Med. 2024 Jan 5;22:26. doi: 10.1186/s12967-023-04846-5 (PMC10768114; doi:10.1186/s12967-023-04846-5)
Supplement: Supplementary file 2 — Additional file 2: Table S1. Parameter values of DKD patients. Table S2. Parameter values of healthy subjects and DKD patients. [file 12967_2023_4846_MOESM2_ESM.docx]

***Supplementary table S1. Parameter values of DKD patients***

| No. | Gender | Age | eGFR  (ml/min/1.73m^2^) | BUN  (mmol/L) | Creatinine  (μmol/L) | UACR  (mg/g) | HbA1c  ( % ) |
| --- | --- | --- | --- | --- | --- | --- | --- |
| 1 | F | 58 | 38 | 12.92 | 139.6 | 5469.82 | 7.7 |
| 2 | F | 42 | 74 | 3.6 | 84 | 266.14 | 6.2 |
| 3 | M | 58 | 40 | 8.23 | 169 | 134.14 | 7.6 |
| 4 | M | 36 | 60 | 4.3 | 95 | 6846.42 | 8.5 |
| 5 | M | 54 | 30 | 8.85 | 220.5 | 8266.96 | 6.9 |
| 6 | F | 45 | 17 | 12 | 279 | 11611.14 | 5.7 |
| 7 | M | 52 | 43 | 11.18 | 164.3 | 4764.95 | 6.5 |
| 8 | M | 52 | 39 | 10.9 | 169 | 11760.16 | 8.5 |
| 9 | F | 41 | 44 | 10.1 | 163 | 4558.03 | 4.3 |
| 10 | M | 28 | 20 | 19.4 | 344 | 6946.68 | 15.4 |
| 11 | M | 54 | 83 | 6.7 | 90 | 1839.84 | 7.8 |
| 12 | F | 58 | 36 | 9.6 | 113 | 16838.34 | 12.5 |
| 13 | F | 62 | 26 | 7.3 | 175 | 10877.69 | 7.4 |
| 14 | M | 57 | 21 | 12.39 | 162 | 7380.64 | 7.8 |
| 15 | F | 49 | 77 | 8.9 | 78 | 6314.36 | 12.9 |
| 16 | M | 47 | 46 | 8.4 | 154 | 5198.08 | 7.5 |

***Supplementary table S2. Parameter values of healthy subjects and DKD patients***

**Parameter values of healthy subjects**

| No. | Gender | Age | Urinary IL-1β  (pg/mg Ucr) | Urinary IL-18  (pg/mg Ucr) | Urinary IL-6  (ng/mg Ucr) |
| --- | --- | --- | --- | --- | --- |
| 1 | F | 66 | 0.122567 | 0.095028 | 208.1288031 |
| 2 | F | 30 | 0.047588 | 0.083966 | 211.2350605 |
| 3 | F | 31 | 0.153502 | 0.019942 | 153.6912473 |
| 4 | M | 45 | 0.043369 | 0.062366 | 441.0950667 |
| 5 | M | 47 | 0.029371 | 0.062028 | 40.25944761 |
| 6 | M | 52 | 0.042346 | 0.000981 | 275.4937656 |
| 7 | F | 18 | 0.028572 | 0.093206 | 1443.264191 |
| 8 | F | 33 | 0.082409 | 0.006413 | 127.0084594 |
| 9 | M | 27 | 0.079008 | 0.060051 | 5.688294846 |
| 10 | M | 40 | 0.036607 | 0.06768 | 27.20031387 |
| 11 | F | 50 | 0.056867 | 0.473477 | undetectable |
| 12 | M | 44 | 0.041009 | 0.439536 | 1080.769757 |
| 13 | M | 48 | 0.03328 | 0.012836 | 341.0820612 |
| 14 | F | 60 | 0.020721 | 0.002035 | undetectable |
| 15 | F | 23 | 0.102726 | 0.03263 | undetectable |
| 16 | M | 48 | 0.055951 | 0.026635 | 43.97124997 |
| 17 | M | 58 | 0.029798 | 0.032587 | 367.885221 |
| 18 | M | 56 | 0.030855 | 0.031819 | 258.184597 |
| 19 | F | 38 | 0.063666 | 0.155694 | 1480.383726 |
| 20 | M | 52 | 0.023338 | 0.004408 | undetectable |
| 21 | F | 50 | 0.055829 | 0.051346 | 290.6319876 |
| 22 | M | 41 | 0.042642 | 0.520796 | 1365.137617 |
| 23 | M | 55 | 0.044918 | 0.083606 | undetectable |

**Parameter values of DKD patients**

| No. | Gender | Age | Urinary IL1β (pg/mg Ucr) | Urinary IL18  (pg/mg Ucr) | Urinary IL6  (ng/mg Ucr) | eGFR  (ml/min/1.73m) | BUN (mmol/L) | Creatinine  (μmol/L) | UACR (mg/g) |
| --- | --- | --- | --- | --- | --- | --- | --- | --- | --- |
| 1 | F | 52 | 7.5237 | 49.335 | 694.6739 | 43 | 11.18 | 164.3 | 4764.95 |
| 2 | M | 62 | 17.1144 | 108.352 | 229.6236 | 95 | 10.2 | 62.8 | 7873.59 |
| 3 | F | 65 | 8.0131 | 895.815 | 2433.904 | 96 | 3.42 | 75.9 | 12973.13 |
| 4 | M | 54 | 9.1108 | 177.894 | 2136.468761 | 46 | 9.23 | 120.4 | 9719.85 |
| 5 | F | 45 | 7.0164 | 427.099 | 3164.878 | 98 | 7.95 | 86 | 8356.9 |
| 6 | F | 48 | 13.3061 | 630.498 | 5070.424 | 10 | 31.4 | 557 | 12227.86 |
| 7 | M | 32 | 6.3277 | 37.413 | 1424.172 | 23 | 14.13 | 205 | 954.06 |
| 8 | M | 50 | 15.553 | 28.121 | 578.3745 | 11 | 12.57 | 419.3 | 8068.01 |
| 9 | F | 54 | 44.7452 | 47.019 | 1225.072 | 47 | 6.46 | 145 | 467.48 |
| 10 | M | 42 | 20.0635 | 1442.2 | 4631.559 | 34 | 13.32 | 163.5 | 5832.64 |
| 11 | M | 52 | 26.4663 | 87.583 | 7752.388 | 63 | 7.3 | 120 | 3508.76 |
| 12 | F | 37 | 3.2876 | 255.306 | 6154.769 | 73 | 7.2 | 87 | 8413.76 |
| 13 | M | 38 | 23.1221 | 30.548 | 37.15327 | 59 | 6.6 | 132 | 446.02 |
| 14 | F | 58 | 5.5786 | 85.554 | 14235.46 | 36 | 9.6 | 113 | 16838.34 |
| 15 | M | 54 | 34.9997 | 305.292 | 2170.469 | 30 | 8.85 | 220.5 | 8266.96 |
| 16 | M | 72 | 83.4645 | 37.485 | 2016.151 | 72 | 6.6 | 96 | 4337.87 |
| 17 | M | 57 | 10.0064 | 494.641 | 4539.13 | 21 | 12.39 | 162 | 7380.64 |
| 18 | F | 43 | 4.2895 | 18.02 | 887.5229077 | 63 | 7.3 | 125 | 224.3 |
| 19 | F | 58 | 38.6522 | 547.339 | 999.3645 | 38 | 12.92 | 139.6 | 5469.82 |
| 20 | F | 62 | 8.3834 | 21.464 | 4545.332 | 57 | 8.41 | 97.2 | 7819.36 |
| 21 | M | 49 | 6.4059 | 80.431 | 2100.101 | 33 | 16.01 | 199 | 6326.96 |
| 22 | F | 34 | 59.3768 | 17.078 | 6248.811 | 15 | 14.61 | 341.4 | 23266.73 |
| 23 | M | 58 | 13.7944 | 1168.284 | 10966.68 | 43 | 11.02 | 125 | 8948.65 |
| 24 | F | 59 | 37.0818 | 460.07 | 2701.067 | 23 | 8.8 | 272 | 6404.33 |
| 25 | F | 69 | 72.488 | 386.3 | 780.2947 | 45 | 8.46 | 138 | 1228.7 |
| 26 | M | 48 | 6.0169 | 178.706 | 759.1137 | 52 | 8.75 | 112.4 | 3758.64 |
| 27 | F | 60 | 11.3458 | 89.415 | 1525.247 | 102 | 7.45 | 62 | 1556.94 |
| 28 | F | 57 | 55.6919 | 35.457 | 1142.281 | 29 | 10.1 | 175 | 2413.23 |
